# Poverty, price and preference barriers to improving diets in sub-Saharan Africa

**DOI:** 10.1016/j.gfs.2022.100664

**Published:** 2023-03

**Authors:** Derek D. Headey, Olivier Ecker, Andrew R. Comstock, Marie T. Ruel

**Affiliations:** International Food Policy Research Institute (IFPRI), 1201 I Street, NW, Washington, DC, 20005, United States

**Keywords:** Healthy diets, Nutritious foods, Food demand, Non-communicable diseases, East Africa

## Abstract

Suboptimal diets are the most important preventable risk factor for the global burden of non-communicable diseases. The EAT-*Lancet* reference diet was therefore developed as a benchmark for gauging divergence from healthy eating standards. However, no previous research has comprehensively explored how and why this divergence exists in poorer countries undergoing nutrition transitions. This study therefore analyzes dietary patterns and drivers of the demand for nutritious foods using nationally representative household surveys from Ethiopia, Kenya, Tanzania, and Uganda. We show how barriers to dietary convergence stem from combinations of poverty, high relative food prices and weak preferences for some specific healthy foods. The article concludes by discussing interventions for strengthening consumer demand for healthy diets in Africa.

## Highlights

• Household data are used to compare diet patterns in Ethiopia, Kenya, Tanzania, and Uganda to the EAT-*Lancet* reference diet.
• Consumption gaps for fruits, vegetables, and pulses and nuts/seeds are large for all consumer groups.
• Food budgets of most households in these countries are far too low to afford the EAT-diet.
• Consumer preferences for vegetables are weak, but preferences for fruits and animal-sourced foods are strong.
• Income growth alone will not solve diet problems in sub-Saharan Africa; both supply and demand side interventions are needed.

## 1 Introduction

Globally, suboptimal diets are one of the most important preventable risk factors for non-communicable diseases (NCDs), accounting for 22% of all deaths and 15% of disability-adjusted life years among adults (Afshin et al., 2019). The dimensions of healthy diets are complex and evidence on the health benefits and costs of consuming specific foods or food groups is imperfect given the high degree of dependence on observational evidence. Even so, there is mounting evidence that some foods and food components significantly elevate the risks of NCDs and associated mortality and morbidity (such as processed red meat, saturated fat, salt, and sugar) while others are protective (such as whole grains, vegetables, fruits, pulses, nuts/seeds and fish). The influential report of the EAT-*Lancet* Commission on healthy diets from sustainable food systems showed that people in most regions of the world overconsume unhealthy foods (e.g. ultra-processed foods rich in sugars, fats, salt and other unhealthy ingredients) and underconsume nutritious foods (e.g., fruits and vegetables), and that a transformation to healthy diets by 2050 will require reducing the global consumption of unhealthy foods by more than 50% and increasing the global consumption of nutritious foods by more than 100% (Willett et al., 2019). While the expected challenges of moving consumers towards consumption of healthy diets are immense, so too are the benefits of preventing early mortality and morbidity from diet-related NCDs and improving the quality of life for millions of people, in addition to achieving substantial environmental benefits (Willett et al., 2019).

Despite these potential benefits, the economic and behavioral challenges of achieving dietary change are still poorly understood, especially in low- and middle-income countries (LMICs). For many households in LMICs, there are likely binding demand-side constraints to increasing consumption of nutritious foods and achieving healthy diets. Incomes and expenditures for many households in LMICs may be lower than the cost of a healthy diet (Headey and Alderman, 2019; Hirvonen et al., 2020). However, for the growing middle-classes, food choices are increasingly driven by time constraints and need for convenience, taste, and social status considerations (Baker and Friel, 2016). LMICs also face significant supply-side constraints stemming from low productivity in the value chains for nutritious foods, especially perishable fresh foods. The union of demand and supply conditions determine the relative prices of different foods, which in turn affect food choices and aggregate costs of nutritionally balanced diets, as well as national food policies and the investment choices of agri-food system actors. Aggressive marketing of ultra-processed foods, for example, plays an important role in shifting consumer preferences (Baker and Friel, 2016).

In this article we compared household food consumption in four East African countries to the healthy reference diet proposed by the EAT-*Lancet* Commission and analyzed the drivers of consumer demand for key food groups in these countries (Willett et al., 2019). We analyzed nationally representative survey data from Ethiopia, Kenya, Tanzania, and Uganda—four countries whose economies and food systems are undergoing rapid transformation. We first show that average East African diets are poorly balanced across major food groups, lacking sufficient amounts of nutritious foods in particular. We then explore three factors that might explain consumption gaps for nutritious foods: poverty (low household food budgets relative to the total cost of the EAT-Lancet reference diet), prices (particularly the high prices of nutritious foods), and preferences (identified by low income elasticities for some nutritious foods). We conclude the article by discussing potential interventions to strengthen consumer demand for nutritious foods and healthy diets in LMICs.

## 2 Methods and materials

### 2.1 Household survey samples and variable construction

The analysis of dietary patterns and drives of the demand for a healthy diet in East Africa uses data from recent household expenditure and consumption surveys from Ethiopia, Kenya, Tanzania, and Uganda. All surveys were conducted between 2014 and 2017 and are representative at the national level and by rural and urban areas. They are the Ethiopia Socioeconomic Survey 2015-16, the Kenya Integrated Household Budget Survey 2015-16, the Tanzania National Panel Survey 2014-15, and the Uganda National Household Survey 2016-17. In all surveys, a 7-day food consumption recall was applied to collect item-level data on food quantities consumed at home, expenditures for purchased foods, and interviewee-estimated “shadow expenditures” (based on sale values) for own-produced and gifted foods.

The main variables used in the analysis were constructed from these food consumption modules: Reported food item consumption quantities were summed up to obtain consumption quantities by food group, after converting non-metric quantity units to metric ones in the cases of Ethiopia and Uganda. They were also converted into item-level calorie consumption amounts (using calorie conversion factors and edible portion coefficients from the *USDA National Nutrient Database* (USDA, 2020) and the *Tanzania Food Composition Table* for some East Africa-specific food items) and summed up to obtain calorie consumption amounts by food group and in total.

Following the same aggregation method, total food expenditures and expenditures by food group were derived from the reported expenditures for purchased items and the estimated value of own-produced and gifted foods. Itemized food prices were approximated from the reported expenditures and consumption quantities only for purchased foods and separately for rural and urban areas of each country. The approximation procedure uses stepwise median calculations at ascending spatial aggregation levels. Food item unit values were averaged at the lowest administrative unit level, if there were at least 10 valid observations for the purchased item (having values larger than 1.5 interquartile range below the 25th percentile value and smaller than 1.5 interquartile range above the 75th percentile value), and if not, the calculation step was repeated at the next higher administrative unit level. The lowest level considered was the “division” (or “county”), followed by the “district” (or “zone”) and then “region.” Household-specific food group prices were derived as the means of the unit values of all consumed food items within the same food group weighted by the household-specific consumption quantity shares of the food items in that food group. Then, a household-specific food price index (used in the food demand system model estimations) was constructed as the mean of food group prices weighted by the consumption quantity shares of the food groups in total food.

Food expenditures and various non-food expenditures, which were reported across different survey modules, were summed up to obtain total household expenditures. Following the method by Deaton and Zaidi to construct consumption aggregates (Deaton and Zaidi, 2002), lumpy infrequent expenditures and tax/levy expenditures were excluded, and “user-cost” rates were applied to obtain the present values for durable goods. The user-cost rate for each durable good reported by a household was calculated based on the good's reported purchase value multiplied by the five-year average of the prevailing interest rate in each country leading up to the time of the survey. Total household expenditure is used as proxy for disposable household income.

Given different household demographics and individual dietary needs, household-level food consumption quantities and calorie consumption amounts were converted to per adult equivalent (AE) to be able to consistently summarize results across households and compare estimates across samples and with the reference intakes of the EAT-Lancet reference diet. An AE expresses an individual household member as a fraction of an adult person—here, in terms of calories. The reference person is an average adult with daily dietary energy requirements of 2500 kcal. This corresponds to the reference intake level of the EAT-Lancet reference diet for total food. Household-specific AE values were calculated from detailed dietary energy requirements for individuals as provided in the FAO/WHO/UNU report (FAO, WHO, & UNU, 2001). These calculations account for household compositions by sex and age and the dietary energy needs of breastfeeding mothers. In the final samples used in the analysis, the average household member corresponds to about 0.88 AE in Tanzania, 0.89 AE in Uganda, 0.90 AE in Ethiopia, and 0.95 AE in Kenya.

Finally, data cleaning was done for consistency across all four surveys, and the procedure included three steps. First, rare, obvious reporting errors in the main variables of the analysis were corrected observation-by-observation. Most of these related to the reported units, such as in the case of implausibly small food quantities that were recorded in kilograms instead of grams, and age of infants recorded in years instead of months. In the second and third steps, entire households were dropped from the samples. Households that did not complete the survey interview, did not report consumption of food at home, or have implausible calorie consumption amounts were dropped. Households were defined as having implausible calorie consumption amounts, if their consumption per AE was below 600 kcal/day or above 6000 kcal/day. Next, for each of the 15 food groups used in the food demand system model estimations, consumption quantities and expenditures were tabulated, and households with implausibly large consumption quantities or expenditures were dropped from the samples.

The final samples used in the analysis included 3249 rural households and 1107 urban households in Ethiopia, 12,318 rural households and 7894 urban households in Kenya, 1871 rural households and 1244 urban households in Tanzania, and 9429 rural households and 4337 urban households in Uganda.

### 2.2 Food demand system model and elasticity calculation

The analysis of the demand drivers for a healthy diet uses complete food demand system models to econometrically estimate parameters that are then used to derive income elasticities of demand for 15 distinct food groups. The model estimations were performed separately by rural and urban areas of each country to allow for structurally different food demand curves. They included two separate estimation stages.

In the first stage, a Working-Leser model (Leser, 1963; Working, 1943) was estimated to derive the income elasticities of total food demand vis-à-vis the aggregate demand for nonfood consumption. This model is conducive to this analysis because it does not require prices for nonfood expenditures that are mostly unobserved in the household surveys used. However, the two-stage approach relies on the assumption of separability between food and nonfood consumption. It is thus assumed that a household first decides on the allocation of the total budget to food and nonfood expenditures and then allocates the food budget to the individual food groups.

Within-food budget allocations were then modeled in the second stage, where full substitutability between all food groups, conditional on the available food budget, is allowed. To estimate food group demand, a quadratic almost ideal demand system (QUAIDS) was used (Banks et al., 1997). The quadratic version was preferred over the more commonly used linear-approximated AIDS (Deaton and Muellbauer, 1980) to allow for the flexibility of a rank-three demand system, which has been shown to be empirically necessary (Buse, 1994; Lewbel, 1991). The standard QUAIDS model specification was augmented to account for censored observations in the dependent variables (food group budget shares of total food expenditure). The two-step procedure used was originally proposed by Shonkwiler and Yen (1999) and later implemented in a QUAIDS framework by Ecker and Qaim (2011). Censoring occurred in the survey data for a considerable number of observations, because households did not consume all 15 food groups during the recall period (of 7 days) but are assumed to do so over a longer observation period. Ignoring censored dependent variables in demand system estimations yields biased parameter estimates. In conformity with the Working-Leser model specification, the standard QUAIDS model is also augmented to control for household economies of scale in food consumption using a linear translation through the intercept.

Assuming weak separability in consumer preferences and low variability of food group prices with income levels, unconditional elasticities were calculated by adding up the conditional elasticities over the two budgeting stages for each household, as suggested by Edgerton (1997). The household-specific income elasticities were averaged at the rural and urban population means in each country, after dropping extreme estimates. As a measure of accuracy of the mean elasticities, standard errors are calculated using a bootstrap estimator. The estimator accounts for clustering at the village/town or city quarter level to account for the correlation among households living in the same neighborhood (Attanasio et al., 2013). The bootstrapping method was performed on the household-specific, conditional income elasticities derived directly from the QUAIDS estimations. The unconditional, mean income elasticities were cleaned for extreme estimates (cutting off the distributions at 1.5 interquartile ranges below the 25th percentile value or above the 75th percentile value).

## 3 Household consumption patterns and healthy reference intakes

**Fig. 1:**
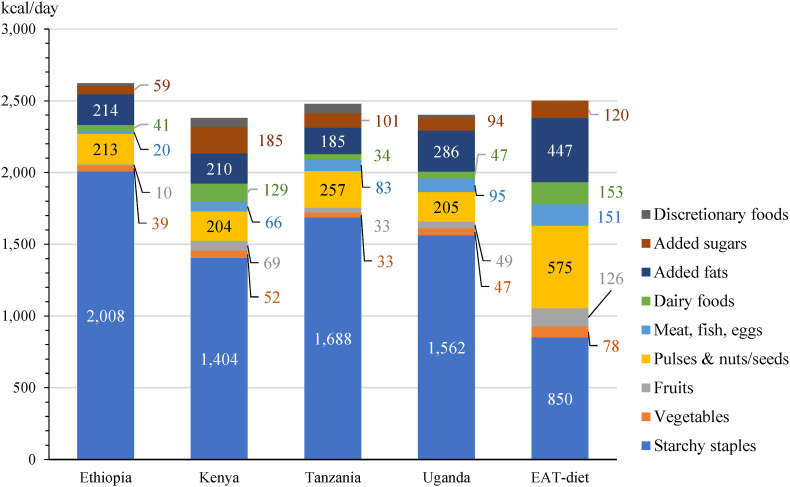
Food consumption calories (kcal/day) per adult-equivalent by major food groups in four sub-Saharan African countries relative to the healthy reference intakes of the EAT-Lancet reference diet Note: One adult equivalent (AE) corresponds to an average adult with a dietary energy requirement of 2500 kcal/day. Consumption estimates refer to foods consumed at home only. Starchy staples include cereals, starchy roots/tubers, and plantains. Discretionary foods include snacks, sweets, and beverages and are considered as non-required foods according to the EAT-Lancet reference diet.

Source: Authors' estimates from nationally representative surveys, in 2014–2017.

Fig. 1 shows average food consumption amounts per adult equivalent (AE) by the major food groups of the EAT-Lancet reference diet, expressed on the basis of calories, in the four East African countries and respective reference intakes of the diet. The EAT-Lancet reference diet consists of a balanced mix of 21 plant-based and animal-source food categories that belong to 7 major food groups. However, we further split the “protein foods” group into those from animal (meat, eggs, fish) and plant sources (legumes, nuts), producing nine food groups. The EAT-Lancet reference diet defines reference intake quantities (with possible intake ranges) for each food category (except for a maximum intake level for added sugars) and provides caloric reference intakes corresponding to these quantities. Reference intakes are expressed for a total caloric intake of about 2500 kilocalories (kcal) per day (which is the daily dietary energy requirement of an average adult), with an allowance of only one third of total calories being derived from starchy staples. While the reference intakes should not be interpreted as strict caloric thresholds that must be achieved, they provide useful benchmarks for a diet that yields sufficient calories from a diverse set of food groups that are likely to also provide adequate amounts of essential macro- and micronutrients for most people. For comparability with the reference intakes, food consumption amounts are scaled to one adult equivalent (AE).

The average diet in these four LMICs has several stark different to the EAT-Lancet reference diet. First, it is likely that many people consume too many calories overall. Average total calorie consumption amounts per AE vary only slightly around 2500 kcal per day (Fig. 1), but these estimates exclude food consumed away from home (that amounts to an estimated 4%–13% of total food expenditures, on average). While inadequate calorie intake still affects some people in these surveys, the overconsumption of energy among a sizable proportion of households is plausible given that all four countries report high prevalence of overweight or obesity among adult women, with national rates varying between 8% and 33% in 2014–2016 (ICF International, 2020).

**Fig. 2:**
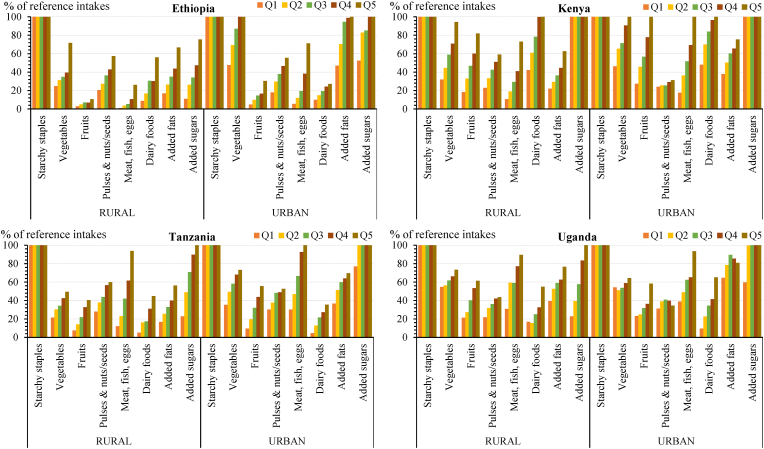
Gaps (%) between estimated household consumption and EAT-Lancet reference diet intakes by expenditure quintile in rural and urban areas Source: Authors' estimates from nationally representative survey data in 2014–2017. Note: Consumption gaps measure the different between consumption quantities and reference intakes, both measured on the basis of calories. Q1 denotes the lowest income quintile by rural or urban areas, and Q5 the richest.

Second, the average diet in these countries is starkly lacking in diversity, with an estimated 59%–77% of total calories stemming from starchy staples and mostly from refined grains whose excess consumption is associated with increased risk of weight gain, metabolic abnormalities, and cardiovascular diseases (see Box 1) (Willett et al., 2019). This dependence on starchy staples means that consumption gaps for healthy food groups are immense, although the size of these gaps varies by household location and economic status, as shown in Fig. 2. Across rural and urban areas of the four countries, the largest differences in the distance to the reference intakes between lower and higher income quintiles exist for the two animal-source food groups (meat/fish/eggs and dairy foods) and fruits. For these food groups, the gaps often exceed 50%, especially among the lowest and middle-income quintiles. However, even the highest quintiles fall short of consuming the reference intakes, except for dairy foods in rural and urban Kenya and meat/fish/eggs in urban areas of Kenya and Tanzania.

Box 1 **Consumption of whole grains and traditional cereals**

The EAT-*Lancet* Commission emphasizes the consumption of whole grains as a central component of a healthy diet. High intake of whole grains has been associated with reduced risk of coronary disease, type 2 diabetes, and overall mortality (Willett et al., 2019). Meanwhile, refined grains have been removed of the grains’ bran, resulting in major loss of micronutrients and fiber. The Commission recommends that carbohydrates should be obtained primarily from whole grains with low intake of refined grains. The EAT-Lancet reference diet suggests an intake level of 232 g/day for an average adult. This is considerably larger than the optimal level of intake (125 g/day) identified in the Global Burden of Disease Study 2017 to minimize the risk from all diet-related causes of death (Afshin et al., 2019).

The bulk of staple crops consumed in Ethiopia, Kenya, and Tanzania are cereals, where they provide 88%–95% of consumed calories from starchy staples, on average. However, cereal consumption in all four countries mainly consists of refined grains and grain products and is dominated by maize flour in Kenya, Tanzania, and Uganda. Cereal consumption is least diverse in Tanzania and Kenya. Maize accounts nationally for 66% (277 g/day per AE) and 63% (238 g/day per AE) of total cereal consumption quantity in Tanzania and Kenya, respectively, of which most comes from the consumption of non-bran maize flours (corresponding to 91% and 79% of total maize consumption, respectively). In Ethiopia, maize consumption is comparably low, accounting for an average of 24% of total cereal consumption quantity (132 g/day per AE). Teff consumption and millet and sorghum consumption average at 27% and 19% of total cereal consumption quantity (146 g/day and 106 g/day per AE), respectively.

In Uganda, cereal consumption is generally low and provides only 34% of all calories obtained from starchy staples, as starchy roots and tubers are the main staple crops. Maize consumption averages 55% of cereal consumption (85 g/day per AE) nationwide; almost all maize is consumed as normal and fine flour (93%). Rice is the second most consumed cereal in Kenya, Tanzania, and Uganda (varying nationally between 29 g/day and 98 g/day per AE and 14% and 23% of total cereal consumption quantities).

Across the four countries, there are notable differences in the consumption of traditional cereals (sorghum, millet, and teff) compared to the Green Revolution cereals (maize, wheat, and rice) between rural and urban areas and, within these areas, between the poor and the rich. The consumed quantities of millet and sorghum are consistently larger and provide greater shares of calories from cereal consumption in rural than urban areas. Within rural and urban areas, millet and sorghum consumption also accounts for larger shares of cereal calories among the lower income quintiles than the higher income quintiles in all four countries. Teff consumption in Ethiopia, however, is much higher in urban than rural areas and among the rich than the poor.

Alt-text: Box 1

Overall, the smallest differences in consumption gaps across income quintiles occur for pulses and nuts/seeds, especially in urban areas, suggesting that demand for this food group does not rise much with total expenditure. In urban Uganda, the average pulse and nut/seed consumption gaps for the richest and poorest income quintiles are somewhat larger than those for the other income quintiles. Average pulse and nut/seed consumption gaps are relatively similar in rural and urban areas, and consumption never exceeds 60% of the reference intake for this food group.

On the other hand, Fig. 2 also shows that, in addition to starchy staples, the two highest income quintiles in urban areas of all four countries overconsume added sugars—even without the inclusion of food consumed away from home. In Kenya, the wealthiest of the four countries, average consumption of sugars among all income quintiles in both urban and rural areas are above the maximum intake level of the EAT-Lancet reference diet.

The consumption breakdown by income quintile also provides some evidence on general consumer preferences for the different food groups. For example, in both rural and urban areas of all four countries, the consumption of animal-source food groups rises sharply with increasing household expenditure levels, as does consumption of fruits, suggesting these are preferred foods. In contrast, vegetable consumption shows variation across geographies, sometimes rising steeply with total expenditure but in some geographies rising very modestly (e.g. Uganda). Notably, pulse and nut/seed consumption increases only marginally with total expenditure in a number of geographies.

## 4 Explaining consumption gaps for healthy foods

What explains the observed large consumption gaps for healthy foods in East Africa? Three key hypotheses are explored: low household food budgets (due to insufficient disposal income), high costs of a healthy diet (due to high prices of nutritious foods in local consumer markets), and weak consumer preferences for healthy foods (as revealed by econometrically derived income elasticities).

### 4.1 The poverty problem: Are household food budgets too low?

**Fig. 3:**
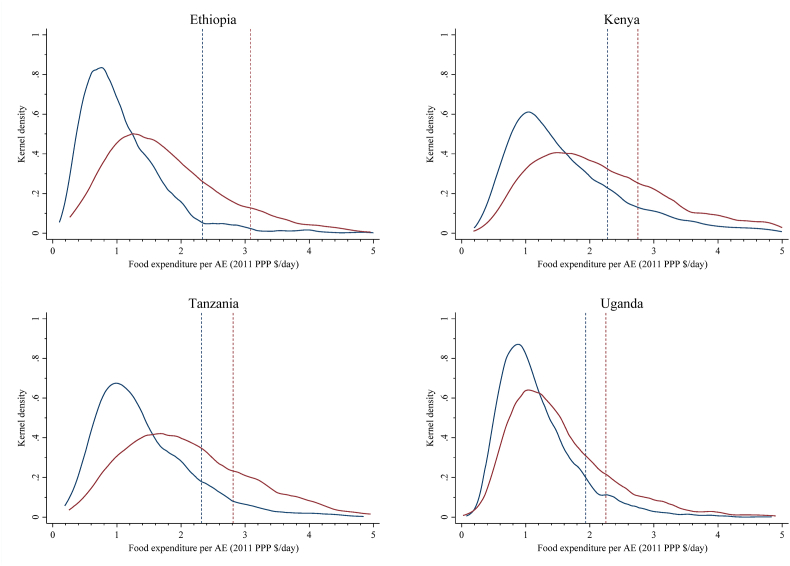
Distributions of household food expenditure and the costs of the EAT-Lancet reference diet in rural areas (blue lines) and urban areas (red lines) Note: One adult equivalent (AE) corresponds to an average adult with a dietary energy requirement of 2500 kcal/day. . (For interpretation of the references to colour in this figure legend, the reader is referred to the Web version of this article.)

Source: Authors' estimates from nationally representative survey data in 2014–2017.

Previous research by Hirvonen and colleagues used nationally averaged price data for 159 countries to demonstrate that the EAT-Lancet reference diet is expensive relative to real household incomes in LMICs (Hirvonen et al., 2020). Here we focus on a more granular comparison of localized EAT-Lancet reference diet costs relative to each household's food expenditures by rural and urban areas of each studied country, graphed in Fig. 3. The cheapest diets that meet the reference intakes from locally sourced foods are always less expensive in rural than urban areas, varying between $1.93 in rural Uganda and $2.33 in rural Ethiopia, while in urban areas it varies between $2.25 in urban Uganda and $3.09 in urban Ethiopia (all in 2011 PPP).

However, consistent with Hirvonen and colleagues’ international analysis, we also find that the majority of households cannot afford the EAT-Lancet reference diet, especially in rural areas where median food expenditure is typically about half of the cost of the cheapest reference diet. Indeed, the shares of the population unable to afford the EAT-Lancet reference diet is extremely high in all four countries, particularly in rural areas: 96% of rural and 91% of urban Ethiopians live in households whose food expenditures are below the costs of the reference diet, while the corresponding rural and urban estimates are 88% and 79% for Kenya, 90% and 80% for Tanzania, and 93% and 87% for Uganda. Thus, small household food budgets are a major barrier towards convergence of current diets to the EAT-Lancet reference diet for most of the population in East Africa.

### 4.2 The price problem: Are the costs of consuming healthy foods too high?

**Fig. 4:**
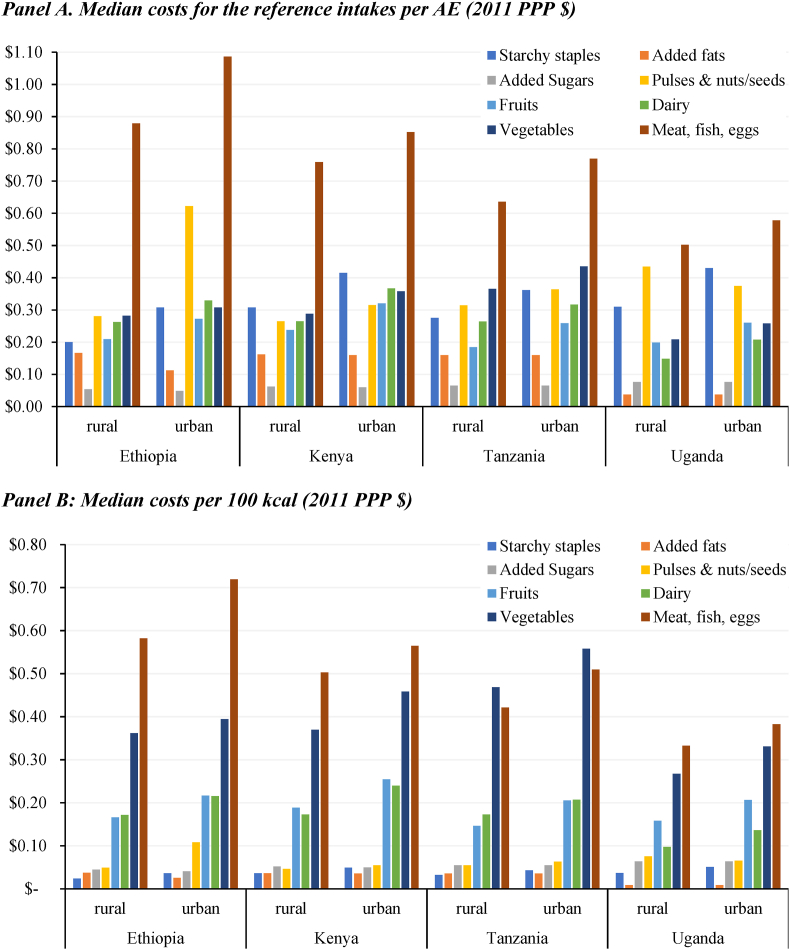
Food group costs of the EAT-Lancet reference diet in rural and urban areas. Note: Reference intakes of the EAT-Lancet reference diet are measured on the basis of calories.

Source: Authors' estimates from nationally representative survey data in 2014–2017.

To assess whether the cost of nutritious foods was a limiting factor in their consumption, we estimated the median costs of the reference intakes per day (Fig. 4, Panel A) and the median costs per 100 Kcal (Panel B) for each required food group of the EAT-Lancet reference diet.

Panel A shows that the meat, fish, and eggs group is the most expensive component of the EAT-Lancet reference diet, costing anywhere between $0.50 and $1.09 per day (in 2011 PPP) to meet recommended intakes, or 26%–38% of the total costs of the EAT-Lancet reference diet. Consuming the reference intakes of other nutritious food groups and starchy staples has broadly similar daily costs of anywhere between $0.15 and $0.43 (except for pulses and nuts/seeds in urban Ethiopia). Yet Panel B shows why food-insecure households would find it difficult to choose foods that are nutrient-dense but expensive sources of calories. The costs per 100 kcal are also the highest for meat, fish, and eggs, ranging from $0.33 and $0.72, followed by vegetables ($0.25 to $0.55 per 100 kcal), and fruits and dairy (both varying between $0.10 and $0.25 per 100 kcal). Pulses, nuts and seeds are always much cheaper than meat/eggs/fish, but costs vary sizably over geographies.

In contrast to these nutrient-dense food groups, staple foods and added fats and sugars are very inexpensive in caloric terms, costing just a few cents per 100 kcal. For very poor and food-insecure households this makes these foods an attractive source of calories and implies that it is economically quite costly for them to diversify away from starchy staples into nutrient dense foods.

### 4.3 The preference problem: Are consumer preferences for nutritious foods too weak?

Fig. 2 indicated that richer households tended to have smaller consumption gaps for EAT-Lancet reference diet food groups than poorer households; clearly, steeper gradients between consumption of a food and total household expenditure would likely imply strong preferences for that food in a given geography. Here we extend that logic by more rigorously estimating income (expenditure) elasticities for different foods as a means of inferring latent food preferences, given relative food prices and various household characteristics, from a demand systems estimation. Specifically, these income elasticities tell us that if a household increases their consumption of a given food by just 3% in response to a 10% increase in income (i.e., an elasticity of approximately 0.3), then it can be inferred that the prevailing preferences for that food is relatively week and that real income growth is unlikely to substantially narrow an existing consumption gap for that food. In contrast elasticities closer to unity would indicate moderately strong preferences, and elasticities in excess of unity would indicate very strong preferences for a given food.

**Fig. 5:**
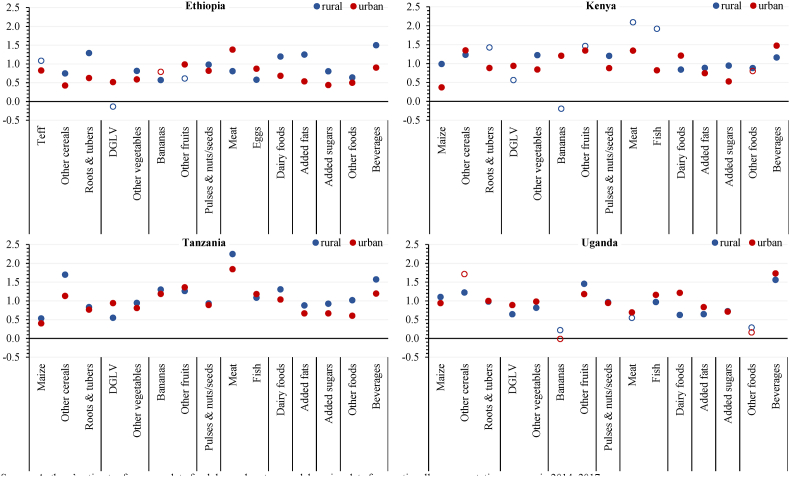
Average income elasticities of food demand in rural and urban areas Notes: Hollow circles denote elasticity estimates that are insignificantly different from zero at the 5% level. DGLV = dark green leafy vegetables. Bananas include plantains. Considering local consumption frequencies, “Eggs” includes fish (rarely consumed) in Ethiopia, but “Fish” includes eggs in Kenya, Tanzania, and Uganda. “Other foods” include snacks, sweets, and other highly processed foods, as well as spices and condiments. . (For interpretation of the references to colour in this figure legend, the reader is referred to the Web version of this article.)

Source: Authors' estimates from complete food demand system models, using data from nationally representative surveys in 2014–2017.

Fig. 5 shows estimated average income elasticities for 15 main food groups, which are a further disaggregation of the major food groups of the EAT-Lancet reference diet implemented to reflect potentially diverse preference for foods within those food groups. For example, we separate bananas (sometimes a staple in Africa) from other fruits, and dark green leafy vegetables from other vegetables. The elasticities are derived from econometric estimates of complete food demand system models for rural and urban areas of each country (see Materials and Methods), and hollow circles represent elasticity estimates that are not statistically significant from zero at the 5% level.

Overall, the results reveal diversity in the revealed consumer preferences across food groups and household locations, but nevertheless present several common food preference patterns across rural and urban areas in the four countries. First, the income elasticities for starchy staples are often surprisingly high, especially in rural areas. This suggests that most consumers in these countries still have relatively strong preferences for staple foods, likely explained by persistent food insecurity and a resulting preference for cheap and storable sources of calories.

Second, the income elasticities for animal-source foods, especially meat, as well as fruits other than bananas are generally high. While meat is an important, nutritious food group especially for children and mothers in poor households, high income responsiveness of meat demand is concerning among wealthier households that already consume enough animal-source foods to support their protein and micronutrient needs. Fish also has high income elasticities in Tanzania and Uganda, where it is an important protein and micronutrient source in rural and urban diets.

Third, income elasticities for vegetables are generally modest in both rural and urban areas of the four countries, suggesting that, with rising income, consumers increase vegetable consumption by lower margins than they do for most animal-source foods. In rural areas, in particular, the income elasticities for dark green leafy vegetables are lower than those for other vegetables, despite being nutrient-dense.

Fourth, income elasticities for pulses and nuts/seeds are close to unity, particularly in rural areas of the four countries, suggesting that rural income growth translates into relatively uniform increases in consumption of pulses and nuts/seeds, which can contribute to narrowing the consumption gaps for these rich plant-based protein and micronutrient sources in rural East Africa. However, income elasticities for pulses are typically much lower than they are for meat in urban geographies.

Finally, income elasticities for potentially unhealthy food groups (including added sugars; other foods such as snacks, sweets, and other highly processed foods; and beverages) consumed at home vary but tend to be quite high, especially for beverages (mainly tea, soft drinks, and alcohol). In addition, separate results (not reported) show that expenditures for food consumed away from home rise at least proportionately with household incomes (especially in urban areas), suggesting that consumer preferences for prepared, possibly unhealthy foods are likely quite strong.

## 5 Discussion

The EAT-*Lancet* Commission report proposed an international healthy reference diet consistent with good health and NCD prevention and further demonstrated that most regions in the world heavily under-consume protective foods and over-consume unhealthy foods (Willett et al., 2019). Economic studies, however, have documented the high costs of nutritious foods (Headey and Alderman, 2019) and healthy diets (Dizon et al., 2019; Mahrt et al., 2019; Raghunathan et al., 2020; FAO, IFAD, UNICEF, WFP, WHO, 2022), including the EAT-diet (Hirvonen et al., 2020). In this study we extended these previous analyses by documenting the large consumption gaps for key nutritious foods among most rural and urban economic groups in four African countries, and systematically exploring why these gaps exist. Low incomes and food expenditures are clearly an overarching constraint in these countries – less than 20% of households can afford the EAT-diet – and poorer and more food-insecure consumers tend to naturally minimize consumption of fruits and vegetables because they are expensive sources of calories (if not of nutrients). However, our demand analysis also reveals instances of low income elasticities for vegetables, which implies that income is not the dominant constraint to increased consumption of these nutritious foods. There are also indications that consumption of pulses, nuts and seeds rises less steeply than it does for animal-sourced foods among better off urban populations. These elasticities imply that even accelerated household income growth over the next decade or two would only close consumption gaps for nutritious foods by a few percentage points. Income elasticities for fruits and animal-sourced foods, on the other hand, are much larger. For some animal sourced foods like meat (red meat especially), which generally has a high income elasticity, rapidly rising incomes can lead to health risks as populations shift to excess consumption (Willett et al., 2019).

This study has several strengths, but also limitations. Diets are consumed by individuals, not households, and aggregation to the household level can introduce recall errors, and ignores intra-household distributional issues in food consumption. However, only household surveys provide nationally representative data for the studied countries, as well as detailed expenditures and food consumption quantities. The surveys in question also vary in terms of how extensive their food lists are, and do not report quantities of food items consumed away from home, so consumption of different foods could be underestimated. The EAT-Lancet reference diet has itself also been questioned for having relatively low and option intake of animal-source foods (Adesogan et al., 2020). Achieving a healthy diet without animal-source foods is possible, although these foods are rich sources of bioavailable essential micronutrients for which deficiencies are common especially among children and women in developing countries. Animal-source foods are also culturally important foods in East Africa. Therefore, this analysis considers animal-source foods as a required food group of the reference diet. An important extension to this kind of research is to use national food-based dietary guidelines instead (Herforth et al., 2019), although these too often fail to separate out meat, fish and eggs from pulses and nuts. Finally, demand system estimation also presents many challenges with household surveys that vary in quality; compelling the researcher to make potentially important choices about the treatment of possible data errors and heterogeneity across different types of households, including farm households that consume some of the food they produce.

Despite these challenges, the main conclusions of our study on the heterogeneous demand for healthy and unhealthy foods is likely to be robust. The significant under-consumption of healthy foods, largely due to affordability issues, is consistent with a global study demonstrating the high relative prices of nutrient-dense foods in Africa (Headey and Alderman, 2019). Africa-wide research on household food demand (Colen et al., 2018) shows strong demand for animal-sourced foods and sugar-sweetened beverages, moderate demand for fruits, and relatively weak demand for pulses, nuts and vegetables. A more nutrition-focused analysis of the dietary patterns of young children in Africa found highly heterogeneous consumption-wealth gradients (Choudhury et al., 2019). Consumption prevalence of dark green leafy vegetables declined with wealth, suggesting very weak demand compared to very strong gradients for fruits, dairy and animal-sourced foods.

Collectively, this body of work suggests potential overconsumption of certain types of nutritious foods as incomes increase even as there are large and potentially persistent gaps for other nutritious foods, such as vegetables and legumes/nuts, that are not income-sensitive. Such heterogeneity in demand suggests the need for multiple types of approaches to achieve healthier diets. Supply-side interventions clearly have some scope to address the high cost and poor accessibility of healthy foods through nutrition-sensitive agricultural programs (Ruel et al., 2018), value chain interventions (Allen and de Brauw, 2018), and cross-cutting investments in efficiency-enhancing infrastructure such as irrigation, electrification, transport and cold storage, and food safety (Muunda et al., 2021). However, the demand analysis in this study also raises concerns that, even as African consumers are increasingly able to afford more nutritious foods and healthy diets, they may not choose to do so. Such findings beg the question of what can be done to foster healthy dietary choices through demand-side interventions.

By far the most common interventions used to affect food choices in LMICs are social behavioral change communications (SBCC) interventions. Personal or group-based BCC interventions, however, have typically focused on improving maternal and child diets and nutrition (Kim et al., 2018), but the approaches used for these nutritionally vulnerable groups may not be cost-effective or easily scaled up or adapted to other demographic groups.

In high income countries, many nutrition-focused interventions have tried to influence the broader consumer population. Interventions in supermarkets are common, including experimental work altering food positioning (An et al., 2013; Huitink et al., 2020; Hyseni et al., 2017) and food labelling (Andreyeva and Luedicke, 2013; Campos et al., 2011; Cecchini and Warin, 2016; Hyseni et al., 2017; Just and Gabrielyan, 2018), both of which show some evidence of impact. However, their applicability to lower income and rural consumers in sub-Saharan Africa is questionable, especially given their limited access to and use of supermarkets. Efforts to affect rural food choice have relied more on production-based interventions, particularly Enhanced Homestead Food Production (EHFP) programs in which agricultural extension (focused on promotion of increasing production of vegetables, fruits, poultry or fish) is combined with SBCC to raise caregiver knowledge and promote greater consumption of nutrient-rich foods, especially mothers and young children (Haselow et al., 2016). These programs show some evidence of impact (An et al., 2013; Huitink et al., 2020; Hyseni et al., 2017), especially when supply-side and demand-side interventions are combined. It is unclear, however, interventions whether there is a need for supply-side interventions where nutritious foods are available and affordable in local markets (Hirvonen and Headey, 2018), what nutritional tradeoffs exist when women adopt new agricultural practices in the context of significant time constraints (Ruel et al., 2018), and whether fruit and vegetable interventions can be implemented in more water-scarce settings (Hirvonen and Headey, 2018). Consideration of water access, market access and women's time constraints is therefore truly critical in the design of EHFP interventions.

A potential alternative or complement to EHFP programs is the use of food/cash transfers that could directly stimulate demand by expanding total food demand and influencing the composition of demand through nutrition BCC. Food/cash transfers have been shown to increase consumption of healthy foods, but also of unhealthy foods in some instances (Ahmed et al., 2019; Almas et al., 2019; Cunha et al., 2018; Hidrobo et al., 2014; Hoddinott & Skoufias, 2004; Leroy et al., 2010). Evidence from Latin America and Egypt (food subsidies) suggests that some of these programs have inadvertently led to rises in overweight/obesity in environments where ultra-processed foods were available, accessible, and affordable (Leroy et al., 2010; Leroy et al., 2019). Potentially, conditional BCC interventions focused on healthy diets and lifestyles for all household members could have synergistic effects with transfers by reducing the risks of both undernutrition and overweight/obesity (Ahmed et al., 2019).

School feeding and take-home-rations (THR) programs have also led to evidence of benefits for child diets as well as cognitive development and schooling outcomes (Aurino et al., 2020; Evans et al., 2012; Gelli et al., 2018; Jomaa et al., 2011; Webb, 2015), and potential spillovers for younger (pre-school) siblings (Aurino et al., 2020). However, there is limited evidence on sustained impacts on diets, few interventions that combine nutritional education with school feeding, and no evidence that we are aware of on whether school-based interventions yield long-term dietary or nutritional benefits through adulthood. One recent study from India did report intergenerational benefits in the form of large height-for-age Z scores among children of mothers exposed to school feeding programs in the 1990s (Chakrabarti et al., 2021). Another recent experimental study from Vietnam combined nutrition education with school snacks and found that students retained improved knowledge after 6 months, but only when education was supported by complementary healthy snacks (Nguyen et al., 2021). Despite limited evidence, the fact that LMICs have extremely young populations, and that long-term food preferences may be formed in childhood and adolescence, suggests that school-based interventions might be an important means of shifting preferences at a generational level.

Finally, it is clear that taxation policies offer scope to alter the relative prices of healthy and unhealthy foods, and hence to potentially improve diets at scale. However, evidence on this issue is solely confined to middle- and high-income countries, and particularly to taxes on unhealthy foods like added sugars and fats, and sugary beverages (Bartlett et al., 2014; Black et al., 2012; Chakrabarti et al., 2018; Eyles et al., 2012; Hyseni et al., 2017; Jou and Techakehakij, 2012; McFadden et al., 2014; Ni Mhurchu et al., 2010; Powell et al., 2013; Sturm et al., 2013; Thow et al. 2014; Waterlander et al., 2012). The ex ante modelling components of this research suggests that taxes will reduce consumption of unhealthy foods, and some empirical evidence from countries like Mexico has also found evidence of impacts on reduced consumption of taxed foods and beverages (Jou and Techakehakij, 2012; Powell et al., 2013; Thow et al., 2014). There is much less evidence on subsidies or vouchers to promote consumption of healthy foods, as well as less practical experience on how these would be implemented in LMICs. In India, the addition of pulses to the nation's Public Distribution Scheme (PDS) has been effective, but pulses are less perishable and logistically less demanding than fresh fruits, vegetables or animal sourced foods (Chakrabarti et al., 2018). In Indonesia, the country's major rice-in-kind program has transitioned to a food voucher scheme designed to diversify diets (*Sembako*). But while early evaluation evidence showed increased consumption of eggs (Banerjee et al., 2021), more anecdotal recent evidence suggests that suppliers for the program have reverted back to rice. Still, interventions to favorably alter the set of food prices facing consumers in principle offers a scalable approach to dietary improvement, provided important logistical challenges can be managed.

In summary, there are many potential points of intervention for improving diets, but a significant dearth of relevant evidence for LMICs, and even less information on cost effectiveness. There are particularly large knowledge gaps on interventions that are likely to have higher potential in LMICs and in remote rural communities, including ICT-based messaging, interventions in wet markets, school-based nutrition education, nutrition education/BCC programs designed to address dietary risks for all household members (not just mothers and young children) and food voucher or public distribution schemes that offer nutrient-dense non-staple foods. Moreover, there is also an urgent need for double-duty interventions that support consumers in achieving healthier diets (through both supply and demand actions) to simultaneously address multiple forms of malnutrition, including overweight/obesity and multiple micronutrient deficiencies (Hawkes et al., 2020).

## Declaration of competing interest

The authors declare the following financial interests/personal relationships which may be considered as potential competing interests: Derek Headey reports financial support was provided by 10.13039/100000877The Rockefeller Foundation. Derek Headey reports financial support was provided by 10.13039/100000865Bill and Melinda Gates Foundation. Marie Ruel reports financial support was provided by 10.13039/100000877The Rockefeller Foundation. Olivier Ecker reports financial support was provided by 10.13039/100000877The Rockefeller Foundation. Andrew Comstock reports financial support was provided by 10.13039/100000877The Rockefeller Foundation.

## Data Availability

The authors do not have permission to share data.
