# Supplementary material for: Poverty, price and preference barriers to improving diets in sub-Saharan Africa
Source: Glob Food Sec. 2023 Mar;36:100664. doi: 10.1016/j.gfs.2022.100664 (PMC10015269; doi:10.1016/j.gfs.2022.100664)
Supplement: Multimedia component 1 [file mmc1.pdf]

Online Supplement “Poverty, price and preference barriers to improving diets in sub-Saharan Africa”

Supplement A: Preparation and analysis of household survey data

This supplement describes the preparation and analysis of the household survey data from the four East African countries, including justifications for some of the key analytical steps.

Calculation of adult equivalents

The descriptive analysis examines food consumption patterns at the household level. Given different household demographics and individual dietary needs, household-level food consumption quantities and calorie consumption amounts are converted to the basis of per adult equivalent (AE) in order to be able to consistently summarize across households and compare estimates across samples and with the recommended intake levels of the EAT reference diets. An AE expresses an individual household member as a fraction of an adult person—here, in terms of daily calorie requirements. The reference adult is an average adult with calorie requirement of 2,500 kcal/day, which corresponds to the recommended calorie intake of the EAT reference diets.

The AE calculation is based on detailed calorie requirement levels for individuals provided by FAO and WHO (2001) and takes account of household compositions by sex and age of the household members and dietary energy needs for breastmilk production (assuming exclusive breastfeeding during the first six months after birth, and thereafter 50% complementary feeding until the end of the first year of life). The calorie requirements for children and adolescents with an average moderate physical activity level (PAL) as reported by FAO and WHO (2001) are used. For adults, calorie requirements are calculated from the formulae based on average PAL and basal metabolic rate (BMR) values, given in the FAO and WHO report. Given the lack of individual physical activity data for the surveyed households, A PAL value of 1.85 is assumed for all adults, which corresponds to a moderately active lifestyle.

An individual's BMR value—the amount of dietary energy used for basal metabolism—depends on her/his body composition. Anthropometric data for adult household members are unavailable from the used surveys, which requires assuming average body weights for the BMR calculations. Sex-age population estimates of mean body heights and mean body-mass-indexes by country are available from the NCD-RisC database, which are used to derive mean body weights that enter the BMR calculations.

In the survey samples used in the analyses, the average household member corresponds to about 0.89 AE in Uganda, 0.90 AE in Ethiopia and Malawi, and 0.95 AE in Kenya.

Data cleaning

Data cleaning is done consistently across all five surveys and includes three steps. First, obvious reporting errors in very few, single observations of the original survey accounts used for the construction of the analysis variables are corrected. Examples include small food quantities recorded in kilograms instead of grams, and age of infants recorded in years instead of months. In the second and third steps, entire households are dropped from the samples. For all food groups used in the demand system estimations, food consumption quantities and expenditures are eyeballed, and households with implausibly large consumption quantities or expenditures are dropped. In the final step, households that did not complete the survey interview, did not report consumption of food at home, or have implausible calorie consumption amounts are dropped. Households are defined as having implausible calorie consumption amounts if their consumption per AE is below 600 kcal/day or above 6,000 kcal/day.

This procedure reduces the household sample sizes by 7% for Kenya and Tanzania, and 12% for Ethiopia and Uganda.

Estimation of income and price elasticities of food demand

The econometric analysis uses a two-stage approach to estimate household-level, unconditional income and Marshallian price elasticities for 15 food groups in a demand system framework. In the first step, a Working-Leser model (Leser 1963; Working 1943) is estimated to derive the elasticities for total food demand. This model is conducive to this analysis because it does not require prices for consumed nonfood goods and services, which are mostly unobserved in the used household survey data. However, the two-stage approach relies on the separability assumption of food and non-food consumption. It is hence assumed that a household first decides on the allocation of the total budget to food and nonfood consumption and then allocates the food budget to the consumption of the 15 food groups.

The food budget allocation is modeled separately in the second stage, where full substitutability between all food groups, conditional on the available food budget, is allowed. A quadratic almost ideal demand system (QUAIDS) is used to estimate food group demands. The quadratic version is chosen rather than the more commonly used linear-approximate AIDS to allow for the flexibility of a rank-three demand system, which has been shown to be empirically necessary (Buse 1994; Lewbel 1991). The standard QUAIDS model specification proposed by Banks et al. (1997) is augmented to account for censored observations in the dependent variables (that are, food group expenditure shares), following the procedure proposed by (Shonkwiler and Yen 1999) and implemented by (Ecker and Qaim 2011), for example. Censoring occurs in the used survey data for a considerable number of observations, because households did not consume all 15 food groups during the recall period (of 7 days) but are assumed to do so over a longer observation period. Ignoring censored dependent variables in demand system estimations yields biased results (Heien and Wessell 1990).

The models are estimated for 10 samples—for rural and urban areas in each studied country. Assuming weak separability in consumer preferences and low variability of food group prices with expenditure levels, unconditional elasticities are derived by adding up the conditional elasticities across the two budgeting stages, as suggested by (Edgerton 1997). Table A2.1-A2.5 show summary statistics of the main variables of the demand system estimations.

Working-Leser model

The estimation equation of the Working-Leser model is:

(1) $$s_h^F = \alpha_0 + \beta \ln M_h + \gamma \ln p_h^F + Z_h' \psi + \varepsilon_h \ ,$$

where h indicates a household. The dependent variable, s_h^F, is the share of total household expenditure spent on food. The main independent variables are total household expenditure (as proxy for household income), M_h, and the household-specific food price index, p_h^F, that enter the estimation equation in logarithms. Income elasticities of total food demand and the aggregate food price elasticities are derived from the estimated coefficients β and γ, respectively. The vector Z_h controls for a standard set of household demographic characteristics, including the AE count per household (in logarithm), and sex, age (in logarithm), and formal education level of the household head, and regional controls at the second administrative level (that is, "districts" in the studied countries).

Standard QUAIDS model

The estimation equations of an AIDS model are a set of budget share equations. The equation system of the standard QUAIDS model (Banks et al., 1997) is given by:

(2) $$w_{hi} = \alpha_i + \beta_i \ln\left[\frac{m_h}{a(p_h)}\right] + \frac{\lambda_i}{b(p_h)}\left\{\ln\left[\frac{m_h}{a(p_h)}\right]\right\}^2 + \sum_{j=1}^{n} \gamma_{ij} \ln p_{hj} \quad \text{for} \quad i = 1, \dots, n \ ,$$

where, in this analysis, w_{hi} is the (latent) household food expenditure share spent on the consumption of food group i (with $n = 15$ and j denoting any food group in the equation system), and m_h is household total food expenditure. Following the original AIDS model formulation by (Deaton and Muellbauer 1980), the translog and Cobb-Douglas price aggregator functions are:

(3) $\ln a(p_h) = \alpha_0 + \sum_{i=1}^{n} \alpha_i \ln p_{hi} + \frac{1}{2}\sum_{i=1}^{n} \sum_{j=1}^{n} \gamma_{ij} \ln p_{hi} \ln p_{hj}$ and

(4) $b(p_h) = \prod_{i=1}^{n} p_{hi}^{\beta_i}$,

where, in this analysis, p_{hi} and p_{hj} are household-specific food group price indices. To complete the QUAIDS specification, the price aggregator function $\lambda(p)$ is defined as:

(5) $\lambda(p) = \sum_{i=1}^{n} \lambda_i \ln p_i$.

In the standard specification, the theoretical restrictions on the parameters of adding-up, homogeneity, and symmetry are imposed on the latent food expenditure shares by:

(6) $\sum_{i=1}^{n} \alpha_i = 1$, $\sum_{i=1}^{n} \beta_i = 0$, $\sum_{i=1}^{n} \lambda_i = 0$, $\sum_{j=1}^{n} \gamma_{ij} = 0$,

(7) $\sum_{i=1}^{n} \gamma_{ji} = 0$, and

(8) $\gamma_{ij} = \gamma_{ji}$ for $i = 1, \dots, n$ and $j = 1, \dots, n$.

Censored QUAIDS with covariates

The standard QUAIDS specification is augmented to account for censored observations in observed household food expenditure shares and to control for household demographic characteristics possibly influencing food budget allocation. (Shonkwiler and Yen 1999)'s procedure to consistently estimate a system of censored food demand equations proceeds in two steps. In this analysis, the first step models whether consumers have access to a particular food group during the observation period and the decision to consume from this food group, and the second step models the decision about the consumption quantity of this food group, considering consumer preferences. The factors determining food access and the decision to consume from a particular food group often differ from the factors that determine how much to consume (Haines et al., 1988).

In the first step, a probit model is applied to obtain the maximum-likelihood probit estimates $\widehat{\omega}_i$ of ω_i for each food group i, using the observed outcome $d_{ih} = 1$, if household h consumed the food group, and $d_{ih} = 0$ otherwise. The binary outcome is regressed on household income (in logarithm); a vector of household demographic characteristics which includes a set of dummy variables that captures household compositions by sex and age groups; sex, age (in logarithm), and formal education level of the household head; and regional controls at the third administrative level (that is, "divisions" or "counties" in the studied countries). These variables together, summarized in the vector X_{hi}, serve to capture household food access and the decision to consume each food group i during the survey recall period, as well as the availability of the items of food group i in the local market at that time. In the second step, the univariate standard normal probability density function $\phi(X_{hi}' \, \widehat{\omega}_i)$ and the cumulative distribution function $\Phi(X_{hi}' \, \widehat{\omega}_i)$ are calculated and included in the estimation equations of the QUAIDS as:

(9) $$w_{hi}^{*} = \Phi(X_{hi}' \,\widehat{\omega}_i)\, w_{hi} + \varphi_i \,\phi(X_{hi}' \,\widehat{\omega}_i)\ ,$$

where w_{hi}^{*} is the latent budget share of food group i. Because of the censoring, the adding-up restriction (Eq. 6) cannot be imposed on the equation system, and the system is correctly estimated by using all n equations (Yen, Kan, and Su 2002).

The estimation equations of the final QUAIDS model are:

(10) $$w_{hi}^{*} = \Phi(X_{hi}' \,\widehat{\omega}_i)\left(\alpha_i + \beta_i \ln\left(\frac{m_h}{a(p_h)}\right) + \frac{\lambda_i}{b(p_h)}\left[\ln\left(\frac{m_h}{a(p_h)}\right)\right]^2 + \sum_{j=1}^{n} \gamma_{ij} \ln p_{hj}\right)$$

$$+ \varphi_i \,\phi(X_{hi}' \,\widehat{\omega}_i) + \psi_i z_h + \varepsilon_{hi} \qquad \text{for} \quad i = 1, \ldots, n\ ,$$

where the covariates z_h denote the household-specific AE counts (in logarithms), capturing household economies of scale in food consumption. Conditional income and Marshallian price elasticities are obtained from the differentials of this set of estimated equations as explained in the next subsection.

Income and price elasticities

Household-specific income elasticities and Marshallian price elasticities of total food demand are obtained from the estimated coefficients of the Working-Leser model as:

(11) $$\epsilon_h^F = \frac{\beta}{w_h^F} + 1 \quad \text{and}$$

(12) $$e_h^F = \frac{\gamma}{w_h^F} - 1\ .$$

Household-specific food group expenditure elasticities and food group price elasticities are derived from the parameters of the QUAIDS by differentiating the set of equations (Eq. 10) with respect to $\ln m_h$ and $\ln p_{hj}$, respectively, as:

(13) $$\mu_{hi} = \Phi(X_{hi}' \,\widehat{\omega}_i)\left(\beta_i + \frac{2\,\lambda_i}{b(p_h)}\left[\ln\left(\frac{m_h}{a(p_h)}\right)\right]\right) \quad \text{and}$$

(14) $\mu_{hij} = \Phi(X'_{hi}\,\widehat{\omega}_i)\left(\gamma_{ij} - \mu_{hi}\left[\alpha_j + \sum_{k=1}^{n}\gamma_{ij}\ln P_k\right] - \frac{\lambda_i\beta_i}{b(p_h)}\left[\ln\left(\frac{m_h}{a(p_h)}\right)\right]^2\right)$,

where P_k is the median price of food group k, averaged across all households within each sample. The conditional expenditure and Marshallian price elasticities of food group consumption for a household h are then obtained by:

(15) $\epsilon_{hi}^{c} = \frac{\mu_{hi}}{w_{hi}^{*}} + 1$ and

(16) $e_{hij}^{c} = \frac{\mu_{hij}}{w_{hi}^{*}} - \delta_{ij}$,

where δ_{ij} is the Kronecker delta equaling one if $i = j$ (yielding own-price elasticities), and zero otherwise (yielding cross-price elasticities). Following (Edgerton 1997), the respective unconditional elasticities are calculated as:

(17) $\epsilon_{hi}^{u} = \epsilon_{h}^{F} * \epsilon_{hi}^{c}$ and

(18) $e_{hij}^{u} = e_{hij}^{c} + \epsilon_{hi}^{c} * w_{hj}^{*}\,(1 + e_{h}^{F})$.

Finally, household-specific income and own- and cross-price elasticities are averaged at the household population means. We used bootstrapped means and standard errors from the uncleaned conditional elasticity estimates to identify income elasticities that were insignificantly different from zero.

Note that Supplement E provides a full set of disaggregated income and own-price elasticities for all geographic and socioeconomic groups.

References on Food Demand Systems Modelling

Buse, Adolf. 1994. "Evaluating the Linearized Almost Ideal Demand System." *American Journal of Agricultural Economics* 76 (4): 781. https://doi.org/10.2307/1243739.

Deaton, Angus, and John Muellbauer. 1980. "An Almost Ideal Demand System." *The American Economic Review* 70 (3): 312–26. https://doi.org/10.1016/0014-2921(94)90008-6.

Deaton, Angus, and Salman Zaidi. 2002. "Guidelines for Constructing Consumption Aggregates For Welfare Analysis." *World Bank*, 1–107. https://doi.org/DOI:

Ecker, Olivier, and Matin Qaim. 2011. "Analyzing Nutritional Impacts of Policies: An Empirical Study for Malawi." *World Development* 39 (3): 412–28. https://doi.org/10.1016/j.worlddev.2010.08.002.

Edgerton, D. L. 1997. "Weak Separability and the Estimation of Elasticities in Multistage Demand Systems." *American Journal of Agricultural Economics* 79 (1): 62–79. https://doi.org/10.2307/1243943.

Heien, Dale, and Cathy Roheirn Wessellc. 1990. "Demand Systems Estimation Reg Rescion Approach" 8 (3).

Leser, Conrad E. 1963. "Form of Engel Functions." *Econometrica* 55 (4): 694–703. https://doi.org/10.2307/1909167.

Lewbel, Arthur. 1991. "The Rank of Demand Systems: Theory and Nonparametric Estimation." *Econometrica* 59 (3): 711. https://doi.org/10.2307/2938225.

Shonkwiler, J. Scott, and Steven T. Yen. 1999. "Two-Step Estimation of a Censored System of Equations." *American Journal of Agricultural Economics* 81 (4): 972. https://doi.org/10.2307/1244339.

Working, Holbrook. 1943. "Statistical Laws of Family Expenditure." *Journal of the American*

Statistical Association 38 (221): 43–56. https://doi.org/10.1080/01621459.1943.10501775.

Yen, Steven T., Kamhon Kan, and Shew Jiuan Su. 2002. "Household Demand for Fats and Oils: Two-Step Estimation of a Censored Demand System." *Applied Economics* 34 (14): 1799–1806. https://doi.org/10.1080/00036840210125008.

Supplement B:

Additional Data on Food Consumption Patterns

Table B1. Average calorie consumption per AE and calorie composition by food groups in study countries, compared to the EAT-Lancet healthy reference diet

	Ethiopia		Kenya		Tanzania		Uganda		Healthy ref. diet	
	Calories (kcal/day)	Share	Calories (kcal/day)	Share	Calories (kcal/day)	Share	Calories (kcal/day)	Share	Calories (kcal/day)	Share
Total food	2,622	100%	2,380	100%	2,461	100%	2,401	100%	2,500	100%
Starchy staples	2,008	77%	1,403	59%	1,685	68%	1,562	65%	850	34%
Vegetables	39	2%	52	2%	20	1%	47	2%	78	3%
Fruits	10	0%	69	3%	33	1%	49	2%	126	5%
Pulses, nuts, seeds	213	8%	204	9%	257	10%	205	9%	575	23%
Meat, fish, eggs	20	1%	66	3%	82	3%	95	4%	151	6%
Dairy foods	41	2%	129	5%	34	1%	47	2%	153	6%
Added fats	214	8%	211	9%	185	8%	286	12%	447	18%
Added sugars	59	2%	185	8%	101	4%	94	4%	120	5%
Snacks & beverages	17	1%	61	3%	65	3%	16	1%	0	0%
Protective foods	262	10%	325	14%	309	13%	302	13%	779	31%
Animal-source foods	61	2%	195	8%	115	5%	141	6%	304	12%

Note: One AE corresponds to an average adult with a calorie requirement of 2,500 kcal/day. Consumption estimates refer to foods consumed at home only. Protective foods include vegetables; fruits; and pulses, nuts, and seeds. Animal-source foods include meat, fish, and eggs and dairy foods. Snacks and (sugar-sweetened and alcoholic) beverages are classified as ‘non-required foods’ by the EAT-Lancet Commission.

Table B2. Average calorie consumption per AE and calorie composition by food groups and residential areas and population strata in Ethiopia, compared to the EAT-Lancet healthy reference diet

	Rural						Urban						Healthy reference diet	
	Total		Poor		Rich		Total		Poor		Rich			
	Calories (kcal/day)	Share	Calories (kcal/day)	Share	Calories (kcal/day)	Share	Calories (kcal/day)	Share	Calories (kcal/day)	Share	Calories (kcal/day)	Share	Calories (kcal/day)	Share
Total food	2,602	100%	1,927	100%	3,320	100%	2,700	100%	2,213	100%	3,254	100%	2,500	100%
Starchy staples	2,060	79%	1,606	83%	2,526	76%	1,807	67%	1,625	73%	2,050	63%	850	34%
Vegetables	31	1%	22	1%	44	1%	70	3%	46	2%	96	3%	78	3%
Fruits	8	0%	5	0%	11	0%	19	1%	10	0%	30	1%	126	5%
Pulses, nuts, seeds	212	8%	140	7%	288	9%	215	8%	142	6%	289	9%	575	23%
Meat, fish, eggs	14	1%	4	0%	29	1%	44	2%	14	1%	85	3%	151	6%
Dairy foods	43	2%	21	1%	67	2%	29	1%	19	1%	39	1%	153	6%
Added fats	169	6%	97	5%	252	8%	388	14%	267	12%	494	15%	447	18%
Added sugars	47	2%	23	1%	76	2%	109	4%	81	4%	138	4%	120	5%
Snacks & beverages	17	1%	8	0%	26	1%	18	1%	8	0%	31	1%	0	0%
Protective foods	252	10%	168	9%	344	10%	305	11%	198	9%	416	13%	779	31%
Animal-source foods	58	2%	24	1%	96	3%	74	3%	33	1%	125	4%	304	12%

Note: One AE corresponds to an average adult with a calorie requirement of 2,500 kcal/day. Consumption estimates refer to foods consumed at home only. Protective foods include vegetables; fruits; and pulses, nuts, and seeds. Animal-source foods include meat, fish, and eggs and dairy foods. Snacks and (sugar-sweetened and alcoholic) beverages are classified as 'non-required foods' by the EAT-Lancet Commission.

The 'poor' are households whose annual household income per capita is in the lowest or second lowest income quintile per residential area. The 'rich' are households whose annual household income per capita is in the highest or second highest income quintile per residential area.

Table B3. Average calorie consumption per AE and calorie composition by food groups and residential areas and population strata in Kenya, compared to the EAT-Lancet healthy reference diet

	Rural						Urban						Healthy reference diet	
	Total		Poor		Rich		Total		Poor		Rich			
	Calories (kcal/day)	Share	Calories (kcal/day)	Share	Calories (kcal/day)	Share	Calories (kcal/day)	Share	Calories (kcal/day)	Share	Calories (kcal/day)	Share	Calories (kcal/day)	Share
Total food	2,423	100%	1,794	100%	2,976	100%	2,322	100%	1,907	100%	2,588	100%	2,500	100%
Starchy staples	1,480	61%	1,188	66%	1,722	58%	1,302	56%	1,198	63%	1,365	53%	850	34%
Vegetables	47	2%	29	2%	63	2%	60	3%	39	2%	73	3%	78	3%
Fruits	60	2%	30	2%	87	3%	80	3%	39	2%	106	4%	126	5%
Pulses, nuts, seeds	240	10%	157	9%	314	11%	156	7%	142	7%	169	7%	575	23%
Meat, fish, eggs	52	2%	21	1%	83	3%	84	4%	32	2%	119	5%	151	6%
Dairy foods	129	5%	76	4%	179	6%	129	6%	80	4%	159	6%	153	6%
Added fats	175	7%	112	6%	235	8%	259	11%	180	9%	306	12%	447	18%
Added sugars	199	8%	161	9%	228	8%	166	7%	161	8%	168	6%	120	5%
Snacks & beverages	42	2%	19	1%	64	2%	88	4%	37	2%	123	5%	0	0%
Protective foods	347	14%	216	12%	464	16%	295	13%	220	12%	348	13%	779	31%
Animal-source foods	181	7%	97	5%	263	9%	213	9%	112	6%	278	11%	304	12%

Note: One AE corresponds to an average adult with a calorie requirement of 2,500 kcal/day. Consumption estimates refer to foods consumed at home only. Protective foods include vegetables; fruits; and pulses, nuts, and seeds. Animal-source foods include meat, fish, and eggs and dairy foods. Snacks and (sugar-sweetened and alcoholic) beverages are classified as 'non-required foods' by the EAT-Lancet Commission.

The 'poor' are households whose annual household income per capita is in the lowest or second lowest income quintile per residential area. The 'rich' are households whose annual household income per capita is in the highest or second highest income quintile per residential area.

Table B4. Average calorie consumption per AE and calorie composition by food groups and residential areas and population strata in Tanzania, compared to the EAT-Lancet healthy reference diet

	Rural						Urban						Healthy reference diet	
	Total		Poor		Rich		Total		Poor		Rich			
	Calories (kcal/day)	Share	Calories (kcal/day)	Share	Calories (kcal/day)	Share	Calories (kcal/day)	Share	Calories (kcal/day)	Share	Calories (kcal/day)	Share	Calories (kcal/day)	Share
Total food	2,425	100%	1,892	100%	2,941	100%	2,535	100%	2,171	100%	2,807	100%	2,500	100%
Starchy staples	1,734	72%	1,486	79%	1,956	67%	1,584	62%	1,485	68%	1,643	59%	850	34%
Vegetables	17	1%	12	1%	21	1%	25	1%	18	1%	31	1%	78	3%
Fruits	29	1%	14	1%	46	2%	40	2%	17	1%	61	2%	126	5%
Pulses, nuts, seeds	260	11%	188	10%	333	11%	250	10%	194	9%	293	10%	575	23%
Meat, fish, eggs	69	3%	26	1%	114	4%	107	4%	56	3%	156	6%	151	6%
Dairy foods	35	1%	16	1%	57	2%	31	1%	13	1%	48	2%	153	6%
Added fats	153	6%	94	5%	214	7%	252	10%	193	9%	302	11%	447	18%
Added sugars	86	4%	43	2%	129	4%	133	5%	111	5%	146	5%	120	5%
Snacks & beverages	42	2%	14	1%	70	2%	113	4%	83	4%	127	5%	0	0%
Protective foods	306	13%	214	11%	400	14%	315	12%	230	11%	385	14%	779	31%
Animal-source foods	104	4%	43	2%	172	6%	138	5%	69	3%	204	7%	304	12%

Note: One AE corresponds to an average adult with a calorie requirement of 2,500 kcal/day. Consumption estimates refer to foods consumed at home only. Protective foods include vegetables; fruits; and pulses, nuts, and seeds. Animal-source foods include meat, fish, and eggs and dairy foods. Snacks and (sugar-sweetened and alcoholic) beverages are classified as 'non-required foods' by the EAT-Lancet Commission.

The 'poor' are households whose annual household income per capita is in the lowest or second lowest income quintile per residential area. The 'rich' are households whose annual household income per capita is in the highest or second highest income quintile per residential area.

Table B5. Average calorie consumption per AE and calorie composition by food groups and residential areas and population strata in Uganda, compared to the EAT-Lancet healthy reference diet

	Rural						Urban						Healthy reference diet	
	Total		Poor		Rich		Total		Poor		Rich			
	Calories (kcal/day)	Share	Calories (kcal/day)	Share	Calories (kcal/day)	Share	Calories (kcal/day)	Share	Calories (kcal/day)	Share	Calories (kcal/day)	Share	Calories (kcal/day)	Share
Total food	2,431	100%	1,925	100%	2,857	100%	2,321	100%	2,055	100%	2,477	100%	2,500	100%
Starchy staples	1,641	68%	1,361	71%	1,853	65%	1,351	58%	1,306	64%	1,376	56%	850	34%
Vegetables	49	2%	44	2%	54	2%	44	2%	41	2%	47	2%	78	3%
Fruits	51	2%	30	2%	71	2%	44	2%	30	1%	59	2%	126	5%
Pulses, nuts, seeds	202	8%	154	8%	247	9%	213	9%	200	10%	216	9%	575	23%
Meat, fish, eggs	95	4%	67	3%	124	4%	93	4%	62	3%	115	5%	151	6%
Dairy foods	44	2%	25	1%	66	2%	53	2%	20	1%	78	3%	153	6%
Added fats	259	11%	204	11%	308	11%	357	15%	299	15%	372	15%	447	18%
Added sugars	76	3%	35	2%	115	4%	141	6%	86	4%	179	7%	120	5%
Snacks & beverages	13	1%	6	0%	21	1%	24	1%	10	0%	36	1%	0	0%
Protective foods	302	12%	227	12%	372	13%	301	13%	271	13%	321	13%	779	31%
Animal-source foods	140	6%	92	5%	189	7%	146	6%	82	4%	193	8%	304	12%

Note: One AE corresponds to an average adult with a calorie requirement of 2,500 kcal/day. Consumption estimates refer to foods consumed at home only. Protective foods include vegetables; fruits; and pulses, nuts, and seeds. Animal-source foods include meat, fish, and eggs and dairy foods. Snacks and (sugar-sweetened and alcoholic) beverages are classified as 'non-required foods' by the EAT-Lancet Commission.

The 'poor' are households whose annual household income per capita is in the lowest or second lowest income quintile per residential area. The 'rich' are households whose annual household income per capita is in the highest or second highest income quintile per residential area.

Supplement C:

Summary statistics for the main variables in the food demand system analysis

Table C1. Summary statistics of the main variables of the demand system estimations for Ethiopia

	Rural (N = 3,249)		Urban (N = 1,106)	
	Mean	SD	Mean	SD
Household expenditure per capita (LCU/day)	18.10	14.95	39.77	29.95
Food expenditure per capita (LCU/day)	13.04	10.09	22.81	14.02
Food expenditure share	0.747	0.157	0.630	0.177
Food group expenditure share for …				
Teff	0.101	0.150	0.216	0.134
Other cereals	0.309	0.215	0.125	0.123
Starchy roots & tubers	0.062	0.127	0.028	0.036
Dark green leafy vegetables	0.015	0.039	0.020	0.035
Other vegetables	0.100	0.096	0.131	0.072
Bananas (incl. plantains)	0.011	0.049	0.017	0.030
Other fruits	0.002	0.017	0.006	0.018
Pulses, nuts, seeds	0.094	0.104	0.102	0.078
Meat	0.046	0.113	0.113	0.150
Fish, eggs, insects	0.010	0.037	0.018	0.035
Dairy foods	0.051	0.096	0.026	0.048
Added fats	0.061	0.071	0.072	0.059
Added sugars	0.023	0.047	0.027	0.028
Snacks & condiments	0.010	0.018	0.006	0.008
Beverages	0.105	0.127	0.093	0.094
Food group consumption (1=yes, 0=no) of …				
Teff	0.442	0.497	0.925	0.264
Other cereals	0.970	0.169	0.917	0.276
Starchy roots & tubers	0.544	0.498	0.823	0.382
Dark green leafy vegetables	0.364	0.481	0.685	0.465
Other vegetables	0.930	0.255	0.993	0.085
Bananas (incl. plantains)	0.183	0.387	0.448	0.498
Other fruits	0.052	0.221	0.154	0.361
Pulses, nuts, seeds	0.736	0.441	0.903	0.296
Meat	0.191	0.393	0.486	0.500
Fish, eggs, insects	0.144	0.351	0.343	0.475
Dairy foods	0.375	0.484	0.399	0.490
Added fats	0.828	0.377	0.945	0.228

Added sugars	0.462	0.499	0.850	0.357
Snacks & condiments	0.938	0.242	0.941	0.235
Beverages	0.837	0.370	0.930	0.255

Table C1—continued.

Food group price (LCU/kg) for …				
Teff	16.57	2.46	17.97	1.64
Other cereals	8.21	2.61	10.69	3.35
Starchy roots & tubers	7.64	2.92	7.82	1.58
Dark green leafy vegetables	5.15	1.17	6.64	1.39
Other vegetables	25.94	25.38	18.50	8.93
Bananas (incl. plantains)	12.33	2.89	14.60	1.13
Other fruits	10.72	5.67	19.08	4.35
Pulses, nuts, seeds	21.43	8.60	32.62	9.78
Meat	103.86	9.82	124.67	9.71
Fish, eggs, insects	44.92	10.67	55.68	3.73
Dairy foods	16.04	3.64	16.86	2.85
Added fats	42.66	24.92	34.70	21.44
Added sugars	20.66	2.23	19.32	0.94
Snacks & condiments	8.85	1.76	8.88	2.40
Beverages	57.61	23.70	55.74	23.47

Table C2. Summary statistics of the main variables of the demand system estimations for Kenya

	Rural (N = 12,318)		Urban (N = 7,894)	
	Mean	SD	Mean	SD
Household expenditure per capita (LCU/day)	169.3	208.7	317.5	383.1
Food expenditure per capita (LCU/day)	80.2	53.5	111.3	70.5
Food expenditure share	0.574	0.187	0.449	0.180
Food group expenditure share for …				
Maize	0.186	0.131	0.116	0.104
Other cereals	0.113	0.095	0.146	0.094
Starchy roots & tubers	0.035	0.054	0.031	0.040
Dark green leafy vegetables	0.013	0.022	0.018	0.023
Other vegetables	0.082	0.066	0.094	0.059
Bananas (incl. plantains)	0.022	0.045	0.026	0.039
Other fruits	0.034	0.057	0.041	0.053
Pulses, nuts, seeds	0.073	0.075	0.050	0.055
Meat	0.077	0.115	0.105	0.109
Fish, eggs, insects	0.035	0.062	0.048	0.071
Dairy foods	0.139	0.119	0.130	0.094
Added fats	0.046	0.041	0.047	0.037
Added sugars	0.082	0.064	0.060	0.048
Snacks & condiments	0.016	0.030	0.025	0.043
Beverages	0.047	0.084	0.064	0.111
Food group consumption (1=yes, 0=no) of …				
Maize	0.976	0.152	0.960	0.196
Other cereals	0.821	0.383	0.920	0.272
Starchy roots & tubers	0.586	0.493	0.683	0.466
Dark green leafy vegetables	0.471	0.499	0.645	0.479
Other vegetables	0.908	0.289	0.975	0.156
Bananas (incl. plantains)	0.424	0.494	0.619	0.486
Other fruits	0.574	0.495	0.715	0.452
Pulses, nuts, seeds	0.810	0.392	0.743	0.437
Meat	0.479	0.500	0.669	0.471
Fish, eggs, insects	0.492	0.500	0.608	0.488
Dairy foods	0.882	0.323	0.901	0.298
Added fats	0.958	0.201	0.962	0.190

Added sugars	0.962	0.191	0.964	0.186
Snacks & condiments	0.980	0.140	0.965	0.184
Beverages	0.945	0.228	0.937	0.243

Table C2—continued.

Food group price (LCU/kg) for …				
Maize	47.7	8.9	50.1	9.1
Other cereals	91.1	14.3	98.8	15.5
Starchy roots & tubers	40.5	19.2	40.8	16.3
Dark green leafy vegetables	34.2	30.7	53.1	59.0
Other vegetables	66.1	37.3	69.8	25.8
Bananas (incl. plantains)	49.6	18.0	54.7	17.4
Other fruits	51.0	19.9	65.0	36.0
Pulses, nuts, seeds	88.0	44.4	97.9	40.6
Meat	357.4	45.7	372.9	46.7
Fish, eggs, insects	278.5	95.4	282.2	93.7
Dairy foods	69.6	80.0	97.8	120.0
Added fats	179.9	40.4	178.9	48.0
Added sugars	98.6	28.7	101.5	32.0
Snacks & condiments	88.3	88.0	112.1	104.7
Beverages	402.2	193.3	376.2	196.2

Table C3. Summary statistics of the main variables of the demand system estimations for Tanzania

	Rural (N = 1,871)		Urban (N = 1,244)	
	Mean	SD	Mean	SD
Household expenditure per capita (LCU/day)	2,120	1,725	4,932	4,195
Food expenditure per capita (LCU/day)	1,230	681	1,871	965
Food expenditure share	0.652	0.173	0.471	0.182
Food group expenditure share for …				
Maize	0.229	0.197	0.108	0.120
Other cereals	0.116	0.137	0.188	0.117
Starchy roots & tubers	0.085	0.110	0.043	0.051
Dark green leafy vegetables	0.038	0.052	0.031	0.033
Other vegetables	0.058	0.049	0.084	0.050
Bananas (incl. plantains)	0.041	0.085	0.036	0.045
Other fruits	0.024	0.051	0.026	0.035
Pulses, nuts, seeds	0.103	0.095	0.083	0.061
Meat	0.091	0.131	0.109	0.109
Fish, eggs, insects	0.081	0.101	0.110	0.097
Dairy foods	0.031	0.069	0.026	0.048
Added fats	0.037	0.031	0.043	0.027
Added sugars	0.030	0.030	0.034	0.022
Snacks & condiments	0.023	0.035	0.049	0.063
Beverages	0.013	0.031	0.029	0.053
Food group consumption (1=yes, 0=no) of …				
Maize	0.893	0.309	0.873	0.333
Other cereals	0.632	0.482	0.928	0.258
Starchy roots & tubers	0.703	0.457	0.757	0.429
Dark green leafy vegetables	0.766	0.424	0.841	0.366
Other vegetables	0.915	0.279	0.982	0.132
Bananas (incl. plantains)	0.358	0.480	0.587	0.493
Other fruits	0.424	0.494	0.658	0.474
Pulses, nuts, seeds	0.839	0.368	0.904	0.295
Meat	0.461	0.499	0.678	0.467
Fish, eggs, insects	0.756	0.430	0.879	0.326
Dairy foods	0.314	0.464	0.363	0.481
Added fats	0.853	0.354	0.958	0.200

Added sugars	0.687	0.464	0.957	0.204
Snacks & condiments	0.996	0.061	0.999	0.028
Beverages	0.580	0.494	0.891	0.311

Table C3—continued.

Food group price (LCU/kg) for …				
Maize	873	177	1,013	115
Other cereals	1,437	212	1,620	240
Starchy roots & tubers	609	176	891	159
Dark green leafy vegetables	808	159	1,046	192
Other vegetables	1,188	144	1,369	153
Bananas (incl. plantains)	875	215	1,232	162
Other fruits	850	210	1,179	177
Pulses, nuts, seeds	1,775	367	1,673	368
Meat	5,253	732	6,211	765
Fish, eggs, insects	3,828	759	4,932	1,169
Dairy foods	1,001	948	1,809	2,443
Added fats	3,026	373	2,927	261
Added sugars	1,962	170	1,890	214
Snacks & condiments	1,045	490	1,775	1,227
Beverages	8,609	3,186	7,220	4,062

Table C4. Summary statistics of the main variables of the demand system estimations for Uganda

	Rural (N = 9,429)		Urban (N = 4,337)	
	Mean	SD	Mean	SD
Household expenditure per capita (LCU/day)	3,534	3,095	6,700	5,887
Food expenditure per capita (LCU/day)	1,656	931	2,163	1,242
Food expenditure share	0.550	0.176	0.417	0.183
Food group expenditure share for …				
Maize	0.101	0.145	0.091	0.125
Other cereals	0.094	0.121	0.132	0.116
Starchy roots & tubers	0.288	0.195	0.213	0.160
Dark green leafy vegetables	0.010	0.020	0.009	0.017
Other vegetables	0.080	0.064	0.078	0.054
Bananas (incl. plantains)	0.007	0.025	0.010	0.030
Other fruits	0.037	0.063	0.033	0.053
Pulses, nuts, seeds	0.138	0.111	0.116	0.092
Meat	0.071	0.110	0.093	0.113
Fish, eggs, insects	0.054	0.080	0.056	0.076
Dairy foods	0.026	0.058	0.036	0.061
Added fats	0.024	0.027	0.031	0.029
Added sugars	0.039	0.049	0.061	0.052
Snacks & condiments	0.012	0.016	0.016	0.027
Beverages	0.019	0.050	0.025	0.060
Food group consumption (1=yes, 0=no) of …				
Maize	0.594	0.491	0.700	0.458
Other cereals	0.636	0.481	0.811	0.392
Starchy roots & tubers	0.901	0.298	0.892	0.310
Dark green leafy vegetables	0.334	0.472	0.361	0.480
Other vegetables	0.962	0.191	0.976	0.152
Bananas (incl. plantains)	0.136	0.343	0.208	0.406
Other fruits	0.514	0.500	0.537	0.499
Pulses, nuts, seeds	0.871	0.335	0.908	0.289
Meat	0.376	0.484	0.510	0.500
Fish, eggs, insects	0.539	0.499	0.612	0.487
Dairy foods	0.304	0.460	0.431	0.495
Added fats	0.700	0.458	0.842	0.365

Added sugars	0.569	0.495	0.821	0.384
Snacks & condiments	0.981	0.138	0.976	0.153
Beverages	0.644	0.479	0.826	0.379

Table C4—continued.

Food group price (LCU/kg) for …				
Maize	2,565	1,688	2,224	675
Other cereals	3,377	1,568	3,816	1,461
Starchy roots & tubers	856	572	892	511
Dark green leafy vegetables	813	252	914	218
Other vegetables	1,162	784	1,451	970
Bananas (incl. plantains)	1,269	383	1,838	567
Other fruits	1,584	839	2,209	1,073
Pulses, nuts, seeds	5,673	4,286	5,133	3,642
Meat	8,873	1,207	9,590	2,810
Fish, eggs, insects	6,295	5,679	6,617	6,696
Dairy foods	1,898	2,181	1,547	1,653
Added fats	2,757	4,686	2,498	3,431
Added sugars	3,977	494	4,057	443
Snacks & condiments	1,555	1,155	1,961	3,571
Beverages	9,430	4,354	10,062	4,194

Supplement D:

Additional results on Food Away From Home (FAFH)

Figure D1. LOWESS regressions of food away from home (FAFH) expenditures as a function of total household expenditure

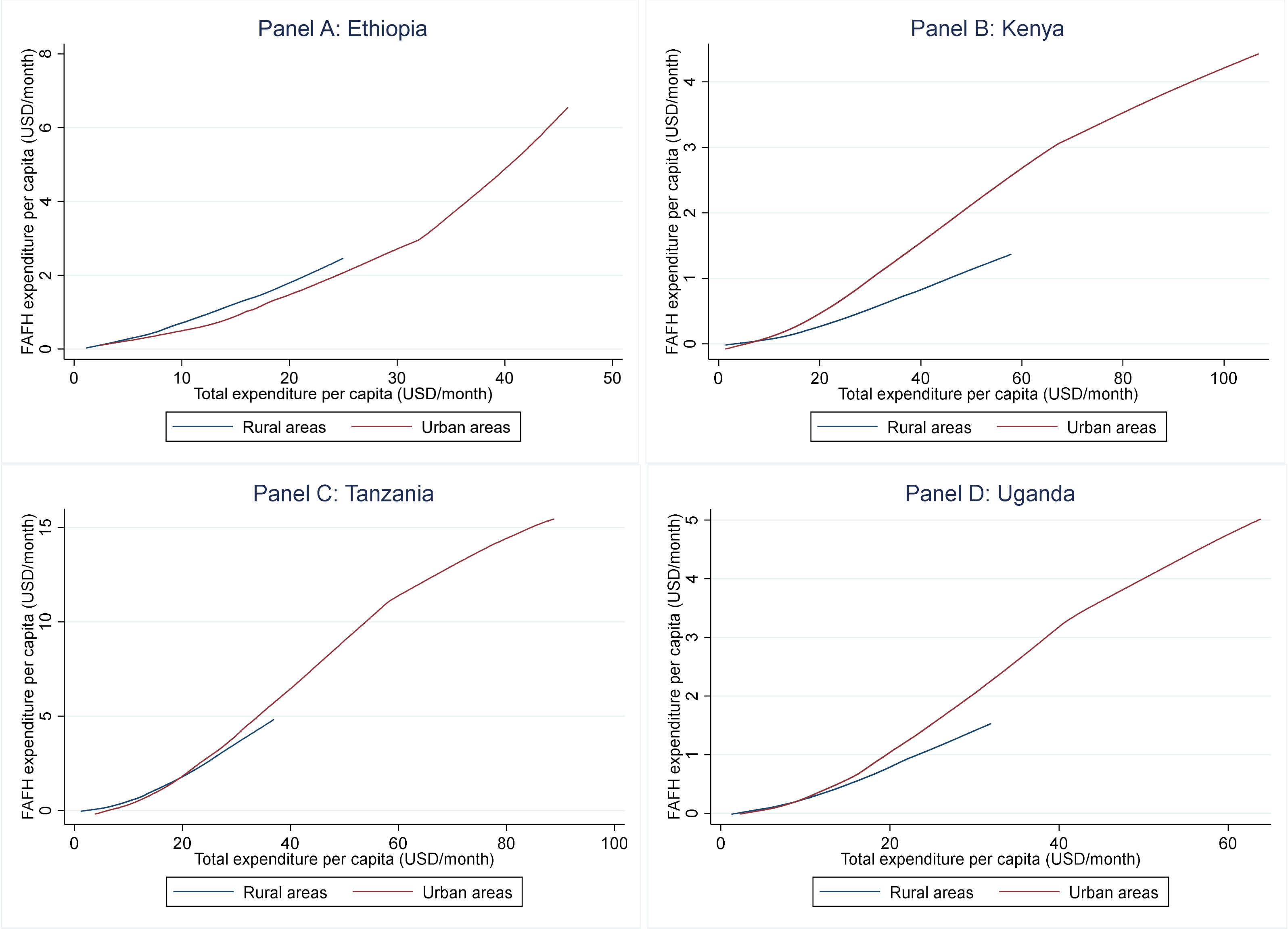

Notes: Total expenditure is capped at the 95th percentile to exclude extreme values.

Figure D2. Food-away-from-home (FAFH) expenditures by expenditure quintiles

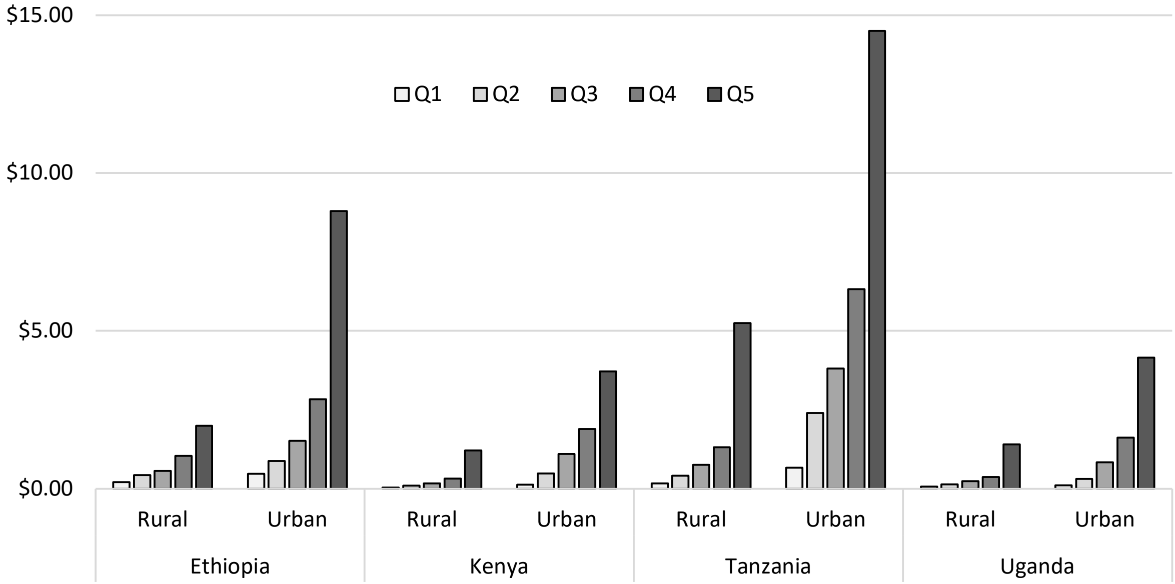

Supplement E. Disaggregated income and own-price elasticities by geography and expenditure quintile

Table E1. Income and own price elasticities in Ethiopia

Expenditure Quintile	Food Group Name	Income elasticity	Own price elasticity
	**RURAL AREAS**		
All	teff/maize	1.08	-1.82
All	other cereals	0.75	-0.85
All	starchy roots & tubers	1.29	-1.24
All	pulses & nuts	0.98	-0.86
All	dairy	1.20	-0.80
All	meat	0.81	-1.55
All	fish & eggs	0.58	-1.78
All	dark green leafy vegetables	-0.14	-0.85
All	other vegetables	0.81	-0.90
All	other fruits	0.61	-0.70
All	bananas & plantains	0.57	-1.22
All	oils & fats	1.25	-0.74
All	sugars	0.80	-0.72
All	condiments & snacks	0.64	-0.52
All	beverages	1.50	-1.03
1	teff/maize	1.62	-2.14
1	other cereals	0.79	-0.87
1	starchy roots & tubers	1.19	-1.29
1	pulses & nuts	1.03	-0.87
1	dairy	1.29	-0.83
1	meat	-0.01	-1.64
1	fish & eggs	0.55	-1.61
1	dark green leafy vegetables	0.07	-0.91
1	other vegetables	0.92	-0.92
1	other fruits	0.19	-0.84
1	bananas & plantains	0.44	-1.17
1	oils & fats	1.11	-0.75
1	sugars	0.81	-0.76
1	condiments & snacks	0.66	-0.58
1	beverages	1.41	-1.08
2	teff/maize	1.42	-1.98
2	other cereals	0.76	-0.85
2	starchy roots & tubers	1.28	-1.27
2	pulses & nuts	1.00	-0.87
2	dairy	1.24	-0.81
2	meat	0.34	-1.69
2	fish & eggs	0.55	-1.73
2	dark green leafy vegetables	-0.13	-0.87
2	other vegetables	0.85	-0.90

2	other fruits	0.32	-0.71
2	bananas & plantains	0.47	-1.21
2	oils & fats	1.21	-0.72
2	sugars	0.80	-0.72
2	condiments & snacks	0.64	-0.53
2	beverages	1.48	-1.04
3	teff/maize	1.02	-1.79
3	other cereals	0.74	-0.84
3	starchy roots & tubers	1.29	-1.23
3	pulses & nuts	0.98	-0.86
3	dairy	1.20	-0.80
3	meat	0.83	-1.64
3	fish & eggs	0.56	-1.83
3	dark green leafy vegetables	-0.22	-0.84
3	other vegetables	0.80	-0.89
3	other fruits	0.63	-0.62
3	bananas & plantains	0.55	-1.23
3	oils & fats	1.27	-0.73
3	sugars	0.80	-0.70
3	condiments & snacks	0.63	-0.51
3	beverages	1.52	-1.02
4	teff/maize	0.54	-1.73
4	other cereals	0.72	-0.83
4	starchy roots & tubers	1.34	-1.23
4	pulses & nuts	0.96	-0.85
4	dairy	1.16	-0.79
4	meat	1.28	-1.49
4	fish & eggs	0.58	-1.88
4	dark green leafy vegetables	-0.29	-0.81
4	other vegetables	0.76	-0.88
4	other fruits	0.97	-0.59
4	bananas & plantains	0.64	-1.24
4	oils & fats	1.32	-0.73
4	sugars	0.80	-0.70
4	condiments & snacks	0.63	-0.50
4	beverages	1.55	-1.02
5	teff/maize	0.76	-1.39
5	other cereals	0.69	-0.82
5	starchy roots & tubers	1.37	-1.19
5	pulses & nuts	0.92	-0.85
5	dairy	1.07	-0.79
5	meat	2.08	-1.21
5	fish & eggs	0.67	-1.95
5	dark green leafy vegetables	-0.20	-0.79

5	other vegetables	0.68	-0.86
5	other fruits	1.24	-0.64
5	bananas & plantains	0.81	-1.26
5	oils & fats	1.36	-0.76
5	sugars	0.79	-0.67
5	condiments & snacks	0.61	-0.45
5	beverages	1.53	-1.01
	**URBAN AREAS**		
All	teff/maize	0.82	-0.71
All	teff/maize	0.82	-0.71
All	other cereals	0.42	-0.87
All	other cereals	0.42	-0.87
All	starchy roots & tubers	0.62	-0.98
All	starchy roots & tubers	0.62	-0.98
All	pulses & nuts	0.82	-0.74
All	pulses & nuts	0.82	-0.74
All	dairy	0.68	-0.78
All	dairy	0.68	-0.78
All	meat	1.38	-0.68
All	meat	1.38	-0.68
All	fish & eggs	0.87	-0.75
All	fish & eggs	0.87	-0.75
All	dark green leafy vegetables	0.51	-0.79
All	dark green leafy vegetables	0.51	-0.79
All	other vegetables	0.59	-0.65
All	other vegetables	0.59	-0.65
All	other fruits	0.98	-0.70
All	other fruits	0.98	-0.70
All	bananas & plantains	0.79	-1.08
All	bananas & plantains	0.79	-1.08
All	oils & fats	0.53	-0.21
All	oils & fats	0.53	-0.21
All	sugars	0.44	-0.61
All	sugars	0.44	-0.61
All	condiments & snacks	0.50	-0.39
All	condiments & snacks	0.50	-0.39
All	beverages	0.90	-1.04
All	beverages	0.90	-1.04
1	teff/maize	1.07	-0.89
1	other cereals	0.33	-0.94
1	starchy roots & tubers	0.75	-0.99
1	pulses & nuts	0.97	-0.77
1	dairy	0.75	-0.80

1	meat	1.01	-0.88
1	fish & eggs	0.96	-0.76
1	dark green leafy vegetables	0.55	-0.80
1	other vegetables	0.66	-0.67
1	other fruits	1.36	-0.69
1	bananas & plantains	1.05	-1.11
1	oils & fats	0.53	-0.27
1	sugars	0.47	-0.70
1	condiments & snacks	0.54	-0.53
1	beverages	0.84	-1.06
2	teff/maize	0.94	-0.79
2	other cereals	0.36	-0.90
2	starchy roots & tubers	0.67	-0.99
2	pulses & nuts	0.88	-0.75
2	dairy	0.71	-0.79
2	meat	1.31	-0.72
2	fish & eggs	0.91	-0.75
2	dark green leafy vegetables	0.54	-0.80
2	other vegetables	0.62	-0.66
2	other fruits	1.13	-0.70
2	bananas & plantains	0.91	-1.09
2	oils & fats	0.53	-0.22
2	sugars	0.44	-0.65
2	condiments & snacks	0.51	-0.44
2	beverages	0.90	-1.05
3	teff/maize	0.79	-0.70
3	other cereals	0.42	-0.85
3	starchy roots & tubers	0.60	-0.98
3	pulses & nuts	0.79	-0.74
3	dairy	0.67	-0.78
3	meat	1.50	-0.61
3	fish & eggs	0.87	-0.74
3	dark green leafy vegetables	0.51	-0.78
3	other vegetables	0.57	-0.64
3	other fruits	0.95	-0.70
3	bananas & plantains	0.76	-1.08
3	oils & fats	0.53	-0.18
3	sugars	0.42	-0.59
3	condiments & snacks	0.49	-0.38
3	beverages	0.92	-1.04
4	teff/maize	0.70	-0.62
4	other cereals	0.49	-0.81
4	starchy roots & tubers	0.54	-0.98
4	pulses & nuts	0.74	-0.73

4	dairy	0.64	-0.76
4	meat	1.60	-0.55
4	fish & eggs	0.83	-0.74
4	dark green leafy vegetables	0.48	-0.77
4	other vegetables	0.54	-0.62
4	other fruits	0.83	-0.70
4	bananas & plantains	0.66	-1.06
4	oils & fats	0.54	-0.20
4	sugars	0.41	-0.54
4	condiments & snacks	0.46	-0.29
4	beverages	0.93	-1.02
5	teff/maize	0.53	-0.52
5	other cereals	0.60	-0.77
5	starchy roots & tubers	0.46	-0.98
5	pulses & nuts	0.65	-0.71
5	dairy	0.59	-0.75
5	meat	1.69	-0.52
5	fish & eggs	0.76	-0.74
5	dark green leafy vegetables	0.47	-0.77
5	other vegetables	0.49	-0.62
5	other fruits	0.63	-0.72
5	bananas & plantains	0.48	-1.05
5	oils & fats	0.55	-0.17
5	sugars	0.42	-0.50
5	condiments & snacks	0.46	-0.27
5	beverages	0.93	-0.99

Table E2. Income and own price elasticities in Kenya

Expenditure Quintile	Food Group Name	Income elasticity	Own price elasticity
	**RURAL AREAS**		
All	maize	0.99	-0.84
All	other cereals	1.23	-0.68
All	starchy roots & tubers	1.42	-1.16
All	pulses & nuts	1.20	-1.06
All	dairy	0.84	-0.62
All	meat	2.09	-0.81
All	fish & eggs	1.92	-0.86
All	dark green leafy vegetables	0.56	-0.84
All	other vegetables	1.22	-1.04
All	other fruits	1.46	-1.07
All	bananas & plantains	-0.20	-0.92
All	oils & fats	0.88	-0.85
All	sugars	0.94	-1.02
All	condiments & snacks	0.88	-0.58
All	beverages	1.16	-1.03
1	maize	1.00	-0.87
1	other cereals	1.51	-0.71
1	starchy roots & tubers	0.95	-1.02
1	pulses & nuts	1.36	-1.05
1	dairy	0.59	-0.62
1	meat	2.13	-0.79
1	fish & eggs	2.31	-0.86
1	dark green leafy vegetables	0.72	-0.80
1	other vegetables	1.32	-1.05
1	other fruits	1.95	-1.03
1	bananas & plantains	-0.05	-0.94
1	oils & fats	0.86	-0.86
1	sugars	0.88	-1.02
1	condiments & snacks	0.91	-0.55
1	beverages	1.17	-1.04
2	maize	0.99	-0.85
2	other cereals	1.38	-0.70
2	starchy roots & tubers	1.78	-1.19
2	pulses & nuts	1.28	-1.05
2	dairy	0.70	-0.59
2	meat	2.33	-0.75
2	fish & eggs	2.49	-0.84

2	dark green leafy vegetables	0.42	-0.79
2	other vegetables	1.27	-1.05
2	other fruits	1.71	-1.06
2	bananas & plantains	-0.22	-0.92
2	oils & fats	0.87	-0.85
2	sugars	0.91	-1.02
2	condiments & snacks	0.89	-0.57
2	beverages	1.16	-1.04
3	maize	0.99	-0.84
3	other cereals	1.26	-0.69
3	starchy roots & tubers	1.69	-1.23
3	pulses & nuts	1.21	-1.06
3	dairy	0.81	-0.58
3	meat	2.20	-0.79
3	fish & eggs	2.08	-0.86
3	dark green leafy vegetables	0.34	-0.81
3	other vegetables	1.23	-1.04
3	other fruits	1.50	-1.07
3	bananas & plantains	-0.32	-0.91
3	oils & fats	0.88	-0.84
3	sugars	0.94	-1.02
3	condiments & snacks	0.88	-0.57
3	beverages	1.16	-1.03
4	maize	0.98	-0.83
4	other cereals	1.13	-0.67
4	starchy roots & tubers	1.57	-1.22
4	pulses & nuts	1.15	-1.06
4	dairy	0.92	-0.62
4	meat	2.07	-0.82
4	fish & eggs	1.80	-0.87
4	dark green leafy vegetables	0.42	-0.86
4	other vegetables	1.19	-1.04
4	other fruits	1.35	-1.08
4	bananas & plantains	-0.29	-0.91
4	oils & fats	0.89	-0.84
4	sugars	0.96	-1.02
4	condiments & snacks	0.87	-0.58
4	beverages	1.16	-1.02
5	maize	0.97	-0.80
5	other cereals	0.94	-0.63
5	starchy roots & tubers	1.11	-1.13
5	pulses & nuts	1.07	-1.06

5	dairy	1.07	-0.70
5	meat	1.86	-0.87
5	fish & eggs	1.40	-0.88
5	dark green leafy vegetables	0.94	-1.00
5	other vegetables	1.13	-1.03
5	other fruits	1.04	-1.09
5	bananas & plantains	-0.11	-0.90
5	oils & fats	0.92	-0.84
5	sugars	1.00	-1.02
5	condiments & snacks	0.85	-0.61
5	beverages	1.15	-1.00
	**URBAN AREAS**		
All	maize	0.37	-0.71
All	other cereals	1.35	-1.09
All	starchy roots & tubers	0.88	-0.65
All	pulses & nuts	0.88	-0.82
All	dairy	1.21	-0.88
All	meat	1.34	-0.76
All	fish & eggs	0.82	-0.34
All	dark green leafy vegetables	0.94	-0.94
All	other vegetables	0.84	-0.84
All	other fruits	1.34	-0.71
All	bananas & plantains	1.20	-1.18
All	oils & fats	0.74	-0.60
All	sugars	0.52	-1.01
All	condiments & snacks	0.80	0.05
All	beverages	1.47	-1.22
1	maize	0.52	-0.79
1	other cereals	1.48	-1.16
1	starchy roots & tubers	0.92	-0.66
1	pulses & nuts	0.96	-0.85
1	dairy	1.20	-0.90
1	meat	1.14	-0.83
1	fish & eggs	0.98	-0.38
1	dark green leafy vegetables	0.79	-0.96
1	other vegetables	0.92	-0.87
1	other fruits	1.18	-0.73
1	bananas & plantains	1.29	-1.15
1	oils & fats	0.86	-0.65
1	sugars	0.66	-1.00
1	condiments & snacks	0.47	0.08

1	beverages	1.71	-1.37
2	maize	0.45	-0.75
2	other cereals	1.42	-1.13
2	starchy roots & tubers	0.90	-0.66
2	pulses & nuts	0.92	-0.84
2	dairy	1.21	-0.89
2	meat	1.25	-0.78
2	fish & eggs	0.89	-0.36
2	dark green leafy vegetables	0.85	-0.95
2	other vegetables	0.88	-0.85
2	other fruits	1.28	-0.70
2	bananas & plantains	1.27	-1.17
2	oils & fats	0.80	-0.63
2	sugars	0.60	-1.00
2	condiments & snacks	0.64	0.08
2	beverages	1.59	-1.30
3	maize	0.39	-0.72
3	other cereals	1.37	-1.10
3	starchy roots & tubers	0.89	-0.65
3	pulses & nuts	0.89	-0.83
3	dairy	1.20	-0.88
3	meat	1.33	-0.75
3	fish & eggs	0.84	-0.35
3	dark green leafy vegetables	0.90	-0.94
3	other vegetables	0.85	-0.84
3	other fruits	1.33	-0.70
3	bananas & plantains	1.24	-1.18
3	oils & fats	0.76	-0.60
3	sugars	0.55	-1.01
3	condiments & snacks	0.73	0.14
3	beverages	1.49	-1.24
4	maize	0.33	-0.69
4	other cereals	1.33	-1.07
4	starchy roots & tubers	0.87	-0.64
4	pulses & nuts	0.86	-0.81
4	dairy	1.21	-0.87
4	meat	1.38	-0.74
4	fish & eggs	0.78	-0.33
4	dark green leafy vegetables	0.96	-0.93
4	other vegetables	0.83	-0.83
4	other fruits	1.37	-0.71
4	bananas & plantains	1.20	-1.19

4	oils & fats	0.72	-0.59
4	sugars	0.49	-1.01
4	condiments & snacks	0.85	0.09
4	beverages	1.43	-1.18
5	maize	0.26	-0.63
5	other cereals	1.25	-1.05
5	starchy roots & tubers	0.85	-0.63
5	pulses & nuts	0.82	-0.80
5	dairy	1.21	-0.86
5	meat	1.44	-0.74
5	fish & eggs	0.72	-0.32
5	dark green leafy vegetables	1.05	-0.92
5	other vegetables	0.78	-0.82
5	other fruits	1.41	-0.73
5	bananas & plantains	1.11	-1.20
5	oils & fats	0.66	-0.55
5	sugars	0.41	-1.02
5	condiments & snacks	0.98	-0.10
5	beverages	1.35	-1.12

Table E3. Income and own price elasticities in Tanzania

Expenditure quintile	Food Group	Income elasticity	Own price elasticity
	**RURAL AREAS**		
All	maize	0.53	-0.76
All	other cereals	1.70	-1.34
All	starchy roots & tubers	0.83	-0.86
All	pulses & nuts	0.93	-0.79
All	dairy	1.31	-0.78
All	meat	2.25	-0.67
All	fish & eggs	1.08	-0.92
All	dark green leafy vegetables	0.55	-0.64
All	other vegetables	0.95	-0.79
All	other fruits	1.26	-0.31
All	bananas & plantains	1.30	-1.02
All	oils & fats	0.88	-0.44
All	sugars	0.92	-0.78
All	condiments & snacks	1.02	-0.29
All	beverages	1.57	-2.98
1	maize	0.67	-0.80
1	other cereals	1.74	-1.32
1	starchy roots & tubers	0.83	-0.88
1	pulses & nuts	1.10	-0.88
1	dairy	1.30	-0.82
1	meat	3.03	-0.32
1	fish & eggs	1.13	-0.93
1	dark green leafy vegetables	0.64	-0.71
1	other vegetables	0.99	-0.81
1	other fruits	1.20	-0.37
1	bananas & plantains	1.28	-1.03
1	oils & fats	1.04	-0.54
1	sugars	0.96	-0.84
1	condiments & snacks	0.95	-0.26
1	beverages	2.07	-2.77
2	maize	0.60	-0.78
2	other cereals	1.70	-1.34
2	starchy roots & tubers	0.83	-0.87
2	pulses & nuts	1.00	-0.82
2	dairy	1.29	-0.80
2	meat	2.54	-0.54
2	fish & eggs	1.11	-0.92

2	dark green leafy vegetables	0.58	-0.66
2	other vegetables	0.97	-0.79
2	other fruits	1.24	-0.32
2	bananas & plantains	1.29	-1.02
2	oils & fats	0.95	-0.48
2	sugars	0.94	-0.80
2	condiments & snacks	0.99	-0.28
2	beverages	1.83	-2.92
3	maize	0.53	-0.76
3	other cereals	1.69	-1.35
3	starchy roots & tubers	0.83	-0.85
3	pulses & nuts	0.93	-0.79
3	dairy	1.31	-0.77
3	meat	2.25	-0.67
3	fish & eggs	1.08	-0.92
3	dark green leafy vegetables	0.53	-0.62
3	other vegetables	0.94	-0.78
3	other fruits	1.26	-0.30
3	bananas & plantains	1.30	-1.02
3	oils & fats	0.87	-0.43
3	sugars	0.93	-0.77
3	condiments & snacks	1.02	-0.29
3	beverages	1.68	-3.07
4	maize	0.44	-0.73
4	other cereals	1.66	-1.35
4	starchy roots & tubers	0.83	-0.84
4	pulses & nuts	0.85	-0.74
4	dairy	1.30	-0.76
4	meat	2.00	-0.80
4	fish & eggs	1.06	-0.91
4	dark green leafy vegetables	0.50	-0.59
4	other vegetables	0.92	-0.77
4	other fruits	1.29	-0.28
4	bananas & plantains	1.31	-1.02
4	oils & fats	0.80	-0.38
4	sugars	0.90	-0.74
4	condiments & snacks	1.05	-0.29
4	beverages	1.33	-3.18
5	maize	0.37	-0.70
5	other cereals	1.69	-1.36
5	starchy roots & tubers	0.83	-0.82
5	pulses & nuts	0.76	-0.69

5	dairy	1.34	-0.72
5	meat	1.87	-0.87
5	fish & eggs	1.04	-0.90
5	dark green leafy vegetables	0.47	-0.56
5	other vegetables	0.90	-0.76
5	other fruits	1.33	-0.27
5	bananas & plantains	1.32	-1.01
5	oils & fats	0.72	-0.32
5	sugars	0.88	-0.71
5	condiments & snacks	1.07	-0.34
5	beverages	1.03	-2.94
	URBAN AREAS		
All	maize	0.40	-0.54
All	other cereals	1.13	-0.87
All	starchy roots & tubers	0.77	-0.71
All	pulses & nuts	0.89	-1.00
All	dairy	1.03	-0.46
All	meat	1.84	-0.73
All	fish & eggs	1.18	-0.91
All	dark green leafy vegetables	0.94	-0.57
All	other vegetables	0.81	-0.66
All	other fruits	1.36	-0.81
All	bananas & plantains	1.19	-0.82
All	oils & fats	0.67	-0.36
All	sugars	0.67	-0.28
All	condiments & snacks	0.60	-0.10
All	beverages	1.19	-2.23
1	maize	0.56	-0.65
1	other cereals	1.16	-0.92
1	starchy roots & tubers	0.90	-0.77
1	pulses & nuts	0.93	-1.01
1	dairy	0.89	-0.58
1	meat	2.03	-0.73
1	fish & eggs	1.10	-0.94
1	dark green leafy vegetables	1.02	-0.62
1	other vegetables	0.86	-0.70
1	other fruits	1.28	-0.82
1	bananas & plantains	1.14	-0.84
1	oils & fats	0.78	-0.48
1	sugars	0.92	-0.50
1	condiments & snacks	0.61	-0.18

1	beverages	1.15	-2.84
2	maize	0.46	-0.58
2	other cereals	1.15	-0.88
2	starchy roots & tubers	0.82	-0.73
2	pulses & nuts	0.90	-1.00
2	dairy	0.97	-0.46
2	meat	1.94	-0.74
2	fish & eggs	1.16	-0.92
2	dark green leafy vegetables	0.97	-0.58
2	other vegetables	0.82	-0.66
2	other fruits	1.35	-0.81
2	bananas & plantains	1.17	-0.82
2	oils & fats	0.71	-0.39
2	sugars	0.78	-0.36
2	condiments & snacks	0.61	-0.13
2	beverages	1.18	-2.34
3	maize	0.39	-0.53
3	other cereals	1.13	-0.87
3	starchy roots & tubers	0.76	-0.70
3	pulses & nuts	0.89	-1.00
3	dairy	1.04	-0.45
3	meat	1.79	-0.74
3	fish & eggs	1.19	-0.91
3	dark green leafy vegetables	0.93	-0.58
3	other vegetables	0.80	-0.65
3	other fruits	1.36	-0.82
3	bananas & plantains	1.19	-0.82
3	oils & fats	0.66	-0.36
3	sugars	0.66	-0.28
3	condiments & snacks	0.61	-0.10
3	beverages	1.23	-2.34
4	maize	0.31	-0.47
4	other cereals	1.12	-0.85
4	starchy roots & tubers	0.71	-0.67
4	pulses & nuts	0.87	-1.00
4	dairy	1.09	-0.42
4	meat	1.75	-0.73
4	fish & eggs	1.20	-0.89
4	dark green leafy vegetables	0.91	-0.55
4	other vegetables	0.78	-0.65
4	other fruits	1.39	-0.81
4	bananas & plantains	1.20	-0.81

4	oils & fats	0.61	-0.29
4	sugars	0.55	-0.16
4	condiments & snacks	0.61	-0.06
4	beverages	1.20	-2.01
5	maize	0.28	-0.42
5	other cereals	1.10	-0.81
5	starchy roots & tubers	0.64	-0.62
5	pulses & nuts	0.86	-1.00
5	dairy	1.14	-0.42
5	meat	1.76	-0.69
5	fish & eggs	1.23	-0.86
5	dark green leafy vegetables	0.88	-0.53
5	other vegetables	0.78	-0.64
5	other fruits	1.41	-0.81
5	bananas & plantains	1.22	-0.80
5	oils & fats	0.58	-0.26
5	sugars	0.43	-0.01
5	condiments & snacks	0.58	-0.02
5	beverages	1.20	-1.80

Table E4. Income and own price elasticities in Uganda

Expenditure quintile	Food Group	Income elasticity	Own price elasticity
	**RURAL AREAS**		
All	maize	1.10	-1.02
All	other cereals	1.22	-1.30
All	starchy roots & tubers	0.98	-0.95
All	pulses & nuts	0.97	-0.91
All	dairy	0.62	-1.39
All	meat	0.55	-0.92
All	fish & eggs	0.97	-1.04
All	dark green leafy vegetables	0.64	-0.76
All	other vegetables	0.81	-1.16
All	other fruits	1.45	-1.10
All	bananas & plantains	0.22	-0.34
All	oils & fats	0.65	-0.79
All	sugars	0.72	-0.52
All	condiments & snacks	0.29	1.20
All	beverages	1.56	-2.16
1	maize	1.12	-1.02
1	other cereals	1.20	-1.25
1	starchy roots & tubers	0.98	-0.96
1	pulses & nuts	0.97	-0.92
1	dairy	0.48	-1.42
1	meat	0.31	-0.90
1	fish & eggs	0.99	-1.05
1	dark green leafy vegetables	0.67	-0.78
1	other vegetables	0.78	-1.14
1	other fruits	1.57	-1.16
1	bananas & plantains	0.15	-0.43
1	oils & fats	0.65	-0.82
1	sugars	0.84	-0.55
1	condiments & snacks	0.26	0.80
1	beverages	1.48	-2.34
2	maize	1.11	-1.02
2	other cereals	1.21	-1.28
2	starchy roots & tubers	0.98	-0.96
2	pulses & nuts	0.97	-0.91
2	dairy	0.46	-1.49
2	meat	0.20	-0.87
2	fish & eggs	0.97	-1.04
2	dark green leafy vegetables	0.64	-0.76
2	other vegetables	0.80	-1.15

2	other fruits	1.48	-1.12
2	bananas & plantains	0.15	-0.34
2	oils & fats	0.65	-0.80
2	sugars	0.77	-0.53
2	condiments & snacks	0.23	1.18
2	beverages	1.60	-2.28
3	maize	1.10	-1.02
3	other cereals	1.21	-1.30
3	starchy roots & tubers	0.98	-0.95
3	pulses & nuts	0.97	-0.91
3	dairy	0.46	-1.50
3	meat	0.40	-0.91
3	fish & eggs	0.97	-1.04
3	dark green leafy vegetables	0.64	-0.75
3	other vegetables	0.81	-1.16
3	other fruits	1.44	-1.10
3	bananas & plantains	0.17	-0.30
3	oils & fats	0.64	-0.79
3	sugars	0.72	-0.52
3	condiments & snacks	0.25	1.35
3	beverages	1.59	-2.15
4	maize	1.09	-1.02
4	other cereals	1.22	-1.31
4	starchy roots & tubers	0.98	-0.95
4	pulses & nuts	0.96	-0.91
4	dairy	0.66	-1.37
4	meat	0.70	-0.96
4	fish & eggs	0.96	-1.04
4	dark green leafy vegetables	0.64	-0.75
4	other vegetables	0.83	-1.17
4	other fruits	1.40	-1.08
4	bananas & plantains	0.22	-0.28
4	oils & fats	0.65	-0.77
4	sugars	0.66	-0.50
4	condiments & snacks	0.32	1.38
4	beverages	1.59	-2.02
5	maize	1.10	-1.02
5	other cereals	1.27	-1.36
5	starchy roots & tubers	0.98	-0.94
5	pulses & nuts	0.96	-0.90
5	dairy	0.86	-1.22
5	meat	1.07	-1.01
5	fish & eggs	0.95	-1.04
5	dark green leafy vegetables	0.64	-0.74

5	other vegetables	0.85	-1.17
5	other fruits	1.39	-1.04
5	bananas & plantains	0.38	-0.32
5	oils & fats	0.64	-0.76
5	sugars	0.64	-0.50
5	condiments & snacks	0.38	1.41
5	beverages	1.56	-1.95
	**URBAN AREAS**		
All	maize	0.94	-0.83
All	other cereals	1.71	-0.54
All	starchy roots & tubers	1.00	-0.98
All	pulses & nuts	0.94	-0.89
All	dairy	1.21	-0.90
All	meat	0.69	-1.30
All	fish & eggs	1.16	-0.90
All	dark green leafy vegetables	0.89	-0.92
All	other vegetables	0.98	-1.06
All	other fruits	1.18	-1.03
All	bananas & plantains	-0.02	-0.52
All	oils & fats	0.83	-0.81
All	sugars	0.71	-0.86
All	condiments & snacks	0.16	1.21
All	beverages	1.73	-2.68
1	maize	0.93	-0.83
1	other cereals	1.50	-0.69
1	starchy roots & tubers	1.02	-1.00
1	pulses & nuts	0.97	-0.91
1	dairy	1.23	-0.91
1	meat	0.70	-1.22
1	fish & eggs	1.19	-0.89
1	dark green leafy vegetables	0.88	-0.92
1	other vegetables	0.87	-1.08
1	other fruits	1.12	-1.02
1	bananas & plantains	-0.08	-0.76
1	oils & fats	0.79	-0.82
1	sugars	0.84	-0.89
1	condiments & snacks	0.20	0.97
1	beverages	1.67	-2.81
2	maize	0.94	-0.83
2	other cereals	1.58	-0.63
2	starchy roots & tubers	1.01	-0.99
2	pulses & nuts	0.96	-0.90
2	dairy	1.22	-0.90

2	meat	0.68	-1.28
2	fish & eggs	1.17	-0.90
2	dark green leafy vegetables	0.88	-0.92
2	other vegetables	0.94	-1.07
2	other fruits	1.16	-1.03
2	bananas & plantains	-0.11	-0.59
2	oils & fats	0.81	-0.81
2	sugars	0.76	-0.87
2	condiments & snacks	0.10	1.28
2	beverages	1.76	-2.82
3	maize	0.94	-0.83
3	other cereals	1.73	-0.52
3	starchy roots & tubers	1.00	-0.98
3	pulses & nuts	0.95	-0.89
3	dairy	1.22	-0.90
3	meat	0.68	-1.30
3	fish & eggs	1.16	-0.90
3	dark green leafy vegetables	0.89	-0.92
3	other vegetables	0.97	-1.07
3	other fruits	1.17	-1.03
3	bananas & plantains	-0.06	-0.52
3	oils & fats	0.82	-0.81
3	sugars	0.71	-0.86
3	condiments & snacks	0.09	1.36
3	beverages	1.72	-2.66
4	maize	0.94	-0.82
4	other cereals	1.83	-0.45
4	starchy roots & tubers	0.99	-0.98
4	pulses & nuts	0.94	-0.89
4	dairy	1.20	-0.91
4	meat	0.70	-1.33
4	fish & eggs	1.15	-0.90
4	dark green leafy vegetables	0.89	-0.92
4	other vegetables	1.01	-1.06
4	other fruits	1.19	-1.03
4	bananas & plantains	0.00	-0.40
4	oils & fats	0.84	-0.81
4	sugars	0.67	-0.85
4	condiments & snacks	0.18	1.21
4	beverages	1.75	-2.67
5	maize	0.94	-0.82
5	other cereals	1.86	-0.40
5	starchy roots & tubers	0.98	-0.97
5	pulses & nuts	0.92	-0.88

5	dairy	1.20	-0.91
5	meat	0.71	-1.34
5	fish & eggs	1.14	-0.90
5	dark green leafy vegetables	0.89	-0.91
5	other vegetables	1.05	-1.04
5	other fruits	1.22	-1.03
5	bananas & plantains	0.12	-0.28
5	oils & fats	0.86	-0.81
5	sugars	0.62	-0.83
5	condiments & snacks	0.23	1.17
5	beverages	1.74	-2.52
